# Construction of stabilized bulk-nano interfaces for highly promoted inverse CeO_2_/Cu catalyst

**DOI:** 10.1038/s41467-019-11407-2

**Published:** 2019-08-02

**Authors:** Han Yan, Chun Yang, Wei-Peng Shao, Li-Hua Cai, Wei-Wei Wang, Zhao Jin, Chun-Jiang Jia

**Affiliations:** Key Laboratory for Colloid and Interface Chemistry, Key Laboratory of Special Aggregated Materials, School of Chemistry and Chemical Engineering, Shandong University, 250100 Jinan, China

**Keywords:** Heterogeneous catalysis, Solid-state chemistry, Catalytic mechanisms

## Abstract

As the water-gas shift (WGS) reaction serves as a crucial industrial process, strategies for developing robust WGS catalysts are highly desiderated. Here we report the construction of stabilized bulk-nano interfaces to fabricate highly efficient copper-ceria catalyst for the WGS reaction. With an in-situ structural transformation, small CeO_2_ nanoparticles (2–3 nm) are stabilized on bulk Cu to form abundant CeO_2_-Cu interfaces, which maintain well-dispersed under reaction conditions. This inverse CeO_2_/Cu catalyst shows excellent WGS performances, of which the activity is 5 times higher than other reported Cu catalysts. Long-term stability is also very solid under harsh conditions. Mechanistic study illustrates that for the inverse CeO_2_/Cu catalyst, superb capability of H_2_O dissociation and CO oxidation facilitates WGS process via the combination of associative and redox mechanisms. This work paves a way to fabricate robust catalysts by combining the advantages of bulk and nano-sized catalysts. Catalysts with such inverse configurations show great potential in practical WGS applications.

## Abstract (web summary)

Cu-CeO_2_ has been considered as promising alternative to Cu-Zn-Al catalyst for water-gas shift (WGS) reaction, but it still suffers from low activity caused by Cu sintering. Here, the authors develop inverse CeO_2_/Cu catalyst with remarkable activity and stability in WGS via construction of stabilized bulk-nano interfaces.

## Introduction

Bulk catalysts with stable structure have been applied to many industrial procedures, such as fused Fe for ammonia synthesis^1^ and Cu–Zn–Al for water–gas shift (WGS) reaction^2^. In recent years, the introduction of nanotechnology has brought valuable insights into heterogeneous catalysis. Remarkable catalytic performances in many reactions have been discovered for supported nanoparticles, clusters, and single atoms^3–7^, of which defects and vacancies on the oxide supports are often considered as important anchoring sites. One major shortcoming of these supported catalysts is the vulnerability against sintering^8–10^. The highly dispersed active sites tend to aggregate and deactivate under reaction conditions. Thus, combining the advantages of bulk and nano-sized catalysts is of great significance, though it seems tough, since the bulk structure contradicts the high dispersion.

WGS (CO + H_2_O = CO_2_ + H_2_) reaction is a crucial process in H_2_ production industry^11^, to which Cu-based catalysts have been applied for decades^12–14^. Cu–CeO_2_ has been considered as promising alternative to Cu–Zn–Al catalyst, while it still suffers from low activity caused by Cu sintering^15–17^. It has been known that the metal–oxide interface plays a critical role in catalyzing WGS reaction, of which the key is the adsorption and activation of reactants^18–20^. To deeply understand the interface effect, catalysts with inverse configuration have been designed^21–23^. Different to commonly supported catalysts, for which active metals are loaded onto the oxide supports, active metals serve as the support for oxides nanoparticles in inverse catalyst^24–27^. Model inverse CeO_*x*_/Cu(111) has shown superior WGS activity to normal Cu/CeO_2_(111), owing to enhanced reducibility of CeO_2_^28^. Meanwhile, the CeO_2_–Cu interface could be highly stable, since bulk Cu has grown well and CeO_2_ nanoparticles are anti-sintering under WGS conditions, which is very beneficial to the catalyst stability. Thus, prominent activity and stability promotion of Cu–CeO_2_ catalyst is expected by applying inverse configuration. However, the assumed high WGS activity has never been found on real CeO_2_/Cu catalyst, due to severe separation of bulk Cu and CeO_2_ nanoparticles^29^. Therefore, strategies to fabricate inverse CeO_2_/Cu catalyst with sufficient bulk–nano interfaces are in great need.

Herein, we have constructed stabilized bulk–nano interfaces to fabricate inverse CeO_2_/Cu catalyst, through which the advantages of bulk and nano-sized catalysts are perfectly combined. With an in situ structural transformation, CeO_2_ nanoparticles (2–3 nm) are dispersed on bulk Cu, forming sufficient CeO_2_–Cu interfaces with great stability. Enrichment of stable bulk–nano interfaces results in great promotion of WGS activity and stability. The inverse CeO_2_/Cu catalyst achieves a remarkable WGS reaction rate of 47.3 μmol g^−1^ s^−1^ (300 °C), which is at least five times of that for other Cu catalysts. Mechanistic study demonstrates the CeO_2_/Cu catalyst possesses superb capability of H_2_O dissociation and CO oxidation, which facilitates WGS reaction via the combination of associative and redox mechanism. Development of such inverse catalyst is very likely to make huge breakthrough in the exploration of other robust catalysts.

## Results

### Catalytic performances of the inverse CeO_2_/Cu catalyst

A series of catalysts with different Cu/Ce ratio was prepared via an aerosol-spray method^30–32^. As shown in Supplementary Fig. 1a and c, the inverse CeO_2_/Cu catalyst with Cu/Ce ratio of 9:1 showed the highest and repeatable WGS activity. Increased or decreased proportion of Cu led to lower CO conversion (Supplementary Fig. 1b). The physical and chemical properties of the catalysts are listed in Supplementary Table 1. Fresh CeO_2_/Cu catalyst contained 61.5 wt% of Cu, and the Cu content elevated to 82.9 wt% after WGS reaction, due to the reduction of CuO to Cu. The 17.1 wt% CeO_2_ loading corresponded well to the finding of Rodriguez et al.^24^, illustrating the optimal CeO_2_ coverage on Cu surface was around 20%. For supported Cu/CeO_2_ catalyst, 10 wt% of Cu has often been applied to obtain an effective catalyst^33–35^. Therefore, normal Cu/CeO_2_ catalyst, with 10.7 wt% of Cu, was chosen for comparison.

**Fig. 1:**
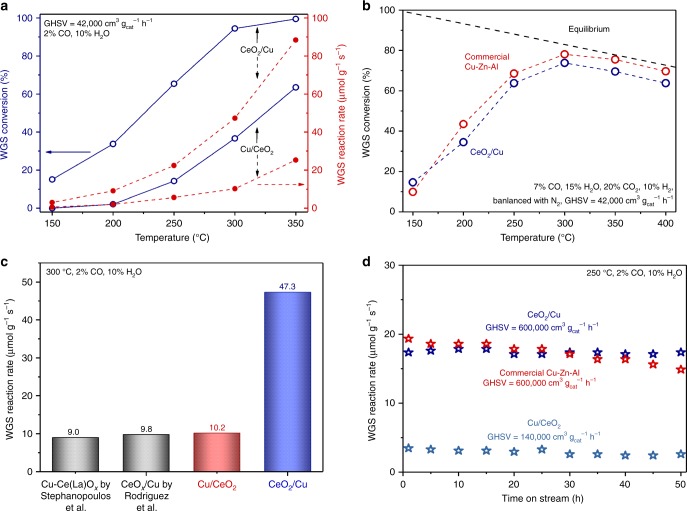
Catalytic performances of the inverse CeO_2_/Cu catalyst. **a** Water–gas shift (WGS) activities of the inverse CeO_2_/Cu and normal Cu/CeO_2_ catalysts. **b** WGS activities of the inverse CeO_2_/Cu catalyst and commercial Cu–Zn–Al under industrial atmosphere. **c** Comparison of WGS reaction rates for different catalysts at 300 °C. **d** Time-on-stream tests of the inverse CeO_2_/Cu, normal Cu/CeO_2_ catalysts, and commercial Cu–Zn–Al

In Table 1, the two fresh catalysts exhibited similar specific BET surface areas (*S*_BET_, 46‒48 m^2^ g^−1^). After catalysis, the *S*_BET_ of CeO_2_/Cu catalyst (16.2 m^2^ g^−1^) apparently decreased, while that of Cu/CeO_2_ catalyst (42.8 m^2^ g^−1^) was well preserved. Cu surface area (*S*_Cu_) was also calculated for both catalysts (see Supplementary Methods for detail). The *S*_Cu_ of Cu/CeO_2_ (71.7 m^2^ g^−1^) was also higher than that of CeO_2_/Cu (50.7 m^2^ g^−1^). However, the inverse CeO_2_/Cu catalyst exhibited much higher WGS conversion than that of Cu/CeO_2_ catalyst (Fig. 1a). Within the tested temperature range, the reaction rate (*r*) of CeO_2_/Cu measured under kinetics conditions was 4–5 times higher than that of Cu/CeO_2_ catalyst. Under the industrial WGS atmosphere, as illustrated in Fig. 1b, the activity of inverse CeO_2_/Cu catalyst was very close to that of commercial Cu–Zn–Al, approaching the equilibrium in harsh reaction conditions. To further demonstrate the promotion of WGS activity, *r* values at 300 °C for different catalysts were illustrated in Fig. 1c. Normal Cu/CeO_2_ gave *r* of 10.2 μmol g^−1^ s^−1^, slightly higher than that of reported Cu–Ce(La)O_*x*_ (9.0 μmol g^−1^ s^−1^)^15^. The similar *r* values here reflected the general WGS activity of supported Cu/CeO_2_ catalysts. Meanwhile, the inverse CeO_2_/Cu gave a very high *r* value of 47.3 μmol g^−1^ s^−1^, which was five times that of normal Cu/CeO_2_ catalyst. Compared to former inverse CeO_*x*_/Cu catalyst (9.8 μmol g^−1^ s^−1^)^29^, the *r* of inverse CeO_2_/Cu was also much higher. The activities of some efficient WGS catalysts are listed in Supplementary Table 2. The inverse CeO_*x*_/Cu catalyst exhibited the highest *r* among the Cu-based catalysts, and its activity was even close to supported Pt catalysts^36,37^. Thus, we believe that the inverse CeO_2_/Cu catalyst with tremendous WGS activity has been successively developed. Arrhenius plots for the catalysts were constructed by using the ln of *r* (Supplementary Fig. 2). In the repeated experiments, the WGS on inverse CeO_2_/Cu gave an apparent energy (*E*_a_) of ca. 37 kJ mol^−1^, which was a little lower than that (ca. 40 kJ mol^−1^) found on normal Cu/CeO_2_.

**Table 1:** Physicochemical properties of fresh and used catalysts

Catalyst	Cu (wt%)^a^		*d*_CeO2_ (nm)^b^		*d*_Cu_ (nm)^b^		*S*_BET_ (m^2^ g^–1^)		*S*_Cu_ (m^2^ g^–1^)	TOF (s^−1^)^c^	Interface sites^c^
CeO_2_/Cu	61.5^d^	82.9^e^	2.6^d^	2.7^e^	8.0^d^	101.2^e^	47.7^d^	16.2^e^	50.7	0.058	4.9 × 10^20^
Cu/CeO_2_	10.7	13.4	3.6	4.7	−	−	46.2	42.8	71.7	0.056	1.1 × 10^20^

^a^Weight ratio of fresh catalysts determined by EDS

^b^CuO for fresh samples, Cu for used samples. Determined by the XRD patterns and Scherrer formula

^c^See detailed calculation process in Supplementary Methods

^d^Data acquired from fresh catalysts

^e^Data acquired from used catalysts

For WGS catalysts, sintering is often considered as the main reason for deactivation^8–10,38^. The well-dispersed active species may aggregate and deactivate rapidly under reaction conditions. Figure 1d present the results of time-on-stream stability tests. Normal Cu/CeO_2_ gave very low *r* (3 μmol g^−1^ s^−1^), while the inverse CeO_2_/Cu exhibited surprisingly high stability. With the temperature of 250 °C and very high space velocity of 600,000 cm^3^ g^−1^ h^−1^, the WGS *r* of the inverse CeO_2_/Cu maintained at 17 μmol g^−1^ s^−1^, showing negligible decrease in the test up to 50 h. The long-term stability of commercial Cu–Zn–Al was measured as well. The initial *r* of Cu–Zn–Al was slightly higher than that of inverse CeO_2_/Cu. After 50 h test, the *r* dropped from 19.5 to 14.8 μmol g^−1^ s^−1^. Thus, the CeO_2_/Cu catalyst gave very solid performance in the time-on-stream test, which showed superior stability under very high space velocity (600,000 cm^3^ g^−1^ h^−1^).

### Construction of bulk–nano interfaces

The catalytic performances were largely determined by the structure of the catalysts. CeO_2_ has been well known as suitable support for Cu catalysts, since the strong interaction between CuO and CeO_2_ could achieve homogeneous Cu dispersion^39–41^. For the fresh CeO_2_/Cu catalyst, such interaction could be confirmed by temperature-programmed reduction by H_2_ (H_2_-TPR), with the fact that reducibility for CuO was enhanced after CeO_2_ addition (Supplementary Fig. 3). Moreover, the strong CuO–CeO_2_ interaction was convinced by the X-ray photoelectron spectroscopy (XPS) (Supplementary Fig. 4a) and ultraviolet–visible spectroscopy (UV–vis) analysis (Supplementary Fig. 5a), showing peak shifting and broadening with increased CeO_2_ content. The strong CuO–CeO_2_ interaction enhanced the redox properties of the catalysts, and improved the dispersion of both Cu and CeO_2_. Raman spectra of the catalysts depicted in Supplementary Fig. 5b exhibited a tiny peak of Raman mode *A*_g_^42,43^, which belonged to cupric oxide. Thus, it seemed difficult to tell whether CeO_2_ was doped into CuO lattice.

**Fig. 2:**
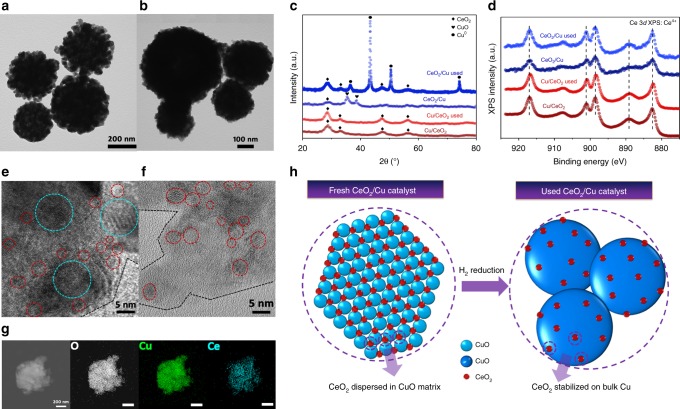
Structure evolution of inverse CeO_2_/Cu catalyst during WGS reaction. **a**, **b** Transmission electron microscope (TEM) images of **a** fresh and **b** used inverse CeO_2_/Cu catalysts. **c** X-ray diffraction (XRD) patterns of the fresh and used catalysts. **d** Ce 3*d* X-ray photoelectron spectroscopy (XPS) results of the fresh and used catalysts. **e**, **f** High-resolution TEM (HR-TEM) images of fresh **e** and used **f** inverse CeO_2_/Cu catalyst, red circles reflect to CeO_2_, blue circles reflect to CuO. **g** Scanning transmission electron microscope (STEM) image and element mapping results of used inverse CeO_2_/Cu catalyst, the inset bar: 200 nm. **h** Scheme of structural evolution for inverse CeO_2_/Cu catalyst during WGS reaction, CeO_2_ nanoparticles are well stabilized despite Cu sintering

The structural and chemical information of inverse CeO_2_/Cu catalyst is given in Fig. 2. The transmission electron microscopy (TEM) image (Fig. 2a) of fresh inverse CeO_2_/Cu catalysts present morphology of microspheres with diameters ranging from 200 to 500 nm. These microspheres were stacked of CuO and CeO_2_ nanoparticles. After WGS reaction, the microspheres became condensed and formed bulk particles (Fig. 2b). Catalysts with other Cu–Ce ratios exhibited similar size and morphology (Supplementary Fig. 6). Powder X-ray diffraction (XRD) results (Supplementary Fig. 7) showed that monoclinic CuO and fluorite CeO_2_ served as dominate phase for the fresh catalyst. In Fig. 2c, the XRD pattern of Cu/CeO_2_ had no observable change after WGS test, indicating the Cu-species remained well-dispersed. This observation correlated well with the stable nature of Cu/CeO_2_ (Fig. 1d). For inverse CeO_2_/Cu catalyst, sharp metallic Cu peaks emerged after WGS test, and the crystalline size of Cu reached 101.2 nm (Table 1). The XRD results were consistent with the trend of dispersity for Cu (Supplementary Table 1), though CeO_2_ has been found to participate in N_2_O chemisorption, causing higher measured dispersion^44^. The XRD data demonstrated that the CeO_2_/Cu catalyst underwent severe sintering and bulk Cu was formed during WGS reaction. However, even though formation of bulk Cu was observed, the WGS activity and long-term stability of inverse CeO_2_/Cu were surprisingly excellent (Fig. 1). This finding was contrary to former knowledge that WGS was favored with smaller Cu-species^38,45^. The unusual phenomenon could be well explained by the fact that WGS reaction occurred at CeO_2_–Cu interfaces of inverse CeO_2_/Cu. As the bulk Cu was present, sufficient interfaces could be created if CeO_2_ was well dispersed. Small CeO_2_ nanoparticles supported on CuO were confirmed by high-resolution TEM (HR-TEM) images in Fig. 2e and Supplementary Fig. 8. After WGS reaction, small-sized CeO_2_ (2–3 nm) was still well-dispersed on bulk Cu (Fig. 2f and Supplementary Fig. 8). The element mapping results (Fig. 2g) further demonstrated the high dispersion of CeO_2_, with Ce signal appearing uniformly on the surface of used CeO_2_/Cu catalyst. XPS profiles of Ce 3*d* were recorded in Fig. 2d. For both inverse and normal catalysts, Ce^4+^ present as the dominating chemical state before and after WGS reaction. The other prepared Cu–CeO_2_ catalysts also exhibited only Ce^4+^ (Supplementary Fig. 4b). Combining with the fact that no shift for the CeO_2_ XRD peaks were observed (Fig. 2c), we believed that the CeO_2_ nanoparticles were supported on CuO, rather than incorporated into the CuO lattice. Therefore, enriched CeO_2_–Cu interfaces were present in the inverse CeO_2_/Cu catalyst.

The structural evolution of the inverse catalyst is depicted in Fig. 2h. With the strong interaction, CeO_2_ nanoparticles were well dispersed in CuO matrix of the fresh sample. After H_2_ reduction, CuO was reduced and sintered to form bulk Cu. Meanwhile, the CeO_2_ nanoparticles showed outstanding stability, holding high dispersion under WGS conditions. The aerosol-spray method enabled the inverse CeO_2_/Cu catalyst to give homogeneous Cu–CeO_2_ dispersion, which induced an in situ structural transformation, resulting in CeO_2_ nanoparticles (2–3 nm) stabilized on bulk Cu. The construction of bulk–nano interfaces brought stable structure of bulk materials and high dispersion of nano-sized catalysts. Tremendous promotion in WGS activity was thus achieved via creation of such bulk–nano interfaces, which was no longer at the risk of sintering deactivation.

### Simulation of sintering via in situ XRD

**Fig. 3:**
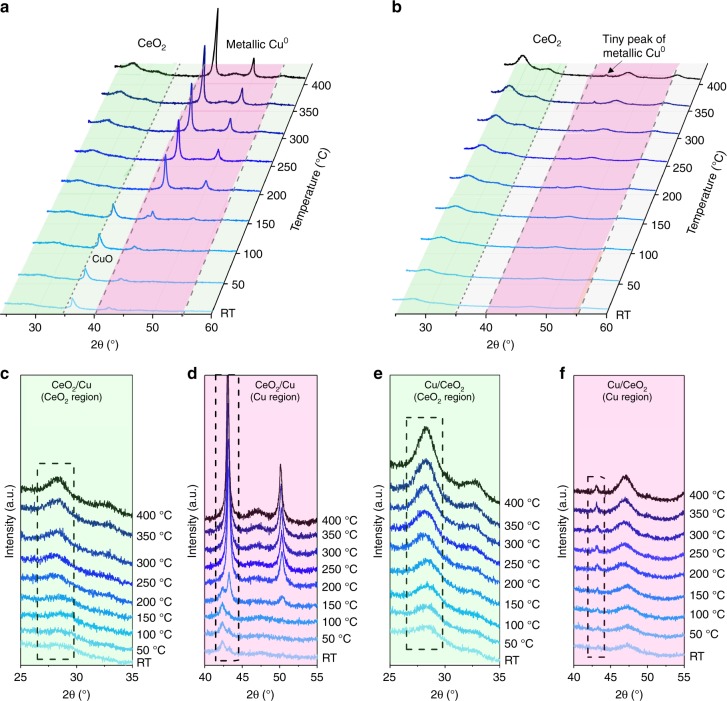
Simulation of sintering via in situ XRD. **a** In situ XRD patterns under 5% H_2_–Ar for inverse CeO_2_/Cu catalyst. **b** In situ XRD patterns under 5% H_2_–Ar for normal Cu/CeO_2_ catalyst. **c** Amplified CeO_2_ region of in situ XRD patterns for inverse CeO_2_/Cu catalyst. **d** Amplified Cu region of in situ XRD patterns for inverse CeO_2_/Cu catalyst. **e** Amplified CeO_2_ region of in situ XRD patterns for normal Cu/CeO_2_ catalyst. **f** Amplified Cu region of in situ XRD patterns for normal Cu/CeO_2_ catalyst

In order to better simulate the sintering phenomenon during catalysis, in situ XRD measurements under 5% H_2_/Ar were performed towards the inverse and normal catalysts. As shown in Fig. 3a, the peaks for metallic Cu of inverse CeO_2_/Cu catalyst emerged at 150 °C and sharpened, indicating rapid sintering of Cu. Amplified Cu region in Fig. 3d illustrates that bulk Cu formed below 200 °C. For normal Cu/CeO_2_ catalyst (Fig. 3b), no obvious Cu peaks could be observed. Amplified Cu region in Fig. 3e displayed a tiny Cu peak centered at 43°. The Cu peak was absent for used Cu/CeO_2_ catalyst (Fig. 2c), which might due to the re-oxidation and dispersion of Cu under ex situ mode. Besides, the structural evolutions of CeO_2_ under reduction are given in Fig. 3c, e. The broad peaks suggested that CeO_2_ nanoparticles in inverse CeO_2_/Cu were maintained at very small size (2.1–2.9 nm, Supplementary Fig. 9), which was in accordance to the XRD data of used inverse catalyst (2.6–2.7 nm, Table 1). Figure 3e shows that the CeO_2_ size of normal Cu/CeO_2_ grew under H_2_ reduction (2.8–5.3 nm, Supplementary Fig. 9). Thus, compared to depositing Cu nanoparticles on CeO_2_ support, the dispersion of CeO_2_ on bulk Cu created more stable CeO_2_–Cu interfaces. This enrichment of stable interfaces for inverse CeO_2_/Cu catalyst resulted in tremendous promotion of WGS activity, corresponding well to former report, in which Cu–CeO_2_ interface was suggested to have great importance in WGS model catalyst^18^.

### WGS mechanism study

For WGS reaction, two catalytic mechanisms have been proposed, namely, redox mechanism and associative mechanism^46–48^. In the redox mechanism, CO reacts with surface oxygen of supports after adsorption, forming CO_2_ and oxygen vacancy. H_2_O dissociates at the vacancy and produces H_2_. In the associative mechanism, CO and H_2_O adsorb on the catalyst to form an intermediate, which decomposes to yield CO_2_ and H_2_. It has been proved that with associative mechanism, surface hydroxyl serves as the active species in Au/CeO_2_ system^49,50^. In this case, two CO molecules react with two surface hydroxyl groups to form 2 CO_2_ molecules and 1 H_2_ molecule:

$$2{\mathrm{CO}} + 2{\mathrm{OH}} = 2\;{\mathrm{CO}}_2 + {\mathrm{H}}_2$$

**Fig. 4:**
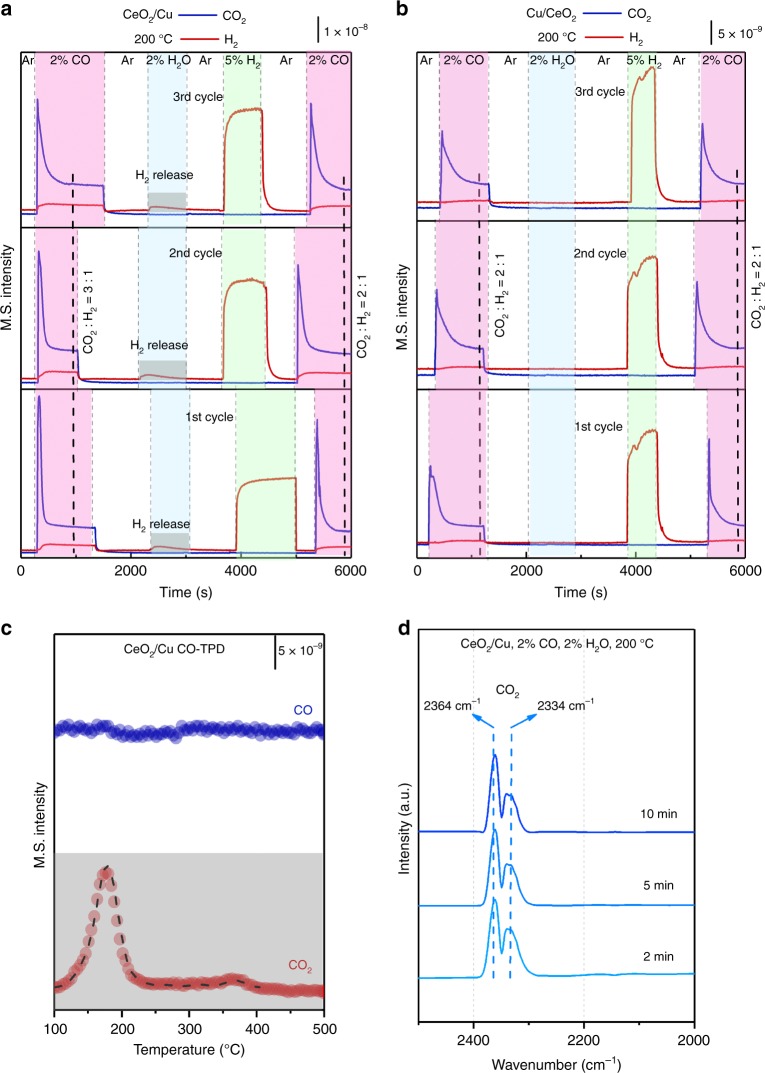
WGS mechanism study of the inverse CeO_2_/Cu catalyst. **a** Temperature-programmed surface reaction (TPSR) on the inverse CeO_2_/Cu with consecutive switch of CO, H_2_O, and H_2_ at 200 °C. **b** TPSR on normal Cu/CeO_2_ with consecutive switch of CO, H_2_O, and H_2_ at 200 °C. **c** Temperature-programmed desorption of CO (CO-TPD) and **d** in situ diffused reflectance infrared Fourier transform spectroscopy (DRIFTS) results of the inverse CeO_2_/Cu under the WGS conditions (2%CO/2%H_2_O/Ar, at 200 °C)

The amount of produced CO_2_ is supposed to be double of that of H_2_ in the outlet gas. Temperature-programmed surface reaction (TPSR) were carried out to check the reaction pathway. As shown in Fig. 4b, CO purging gave the ratio of generated CO_2_ and H_2_ as 2:1, corresponding very well to the above reaction. After H_2_O was introduced, no H_2_ signal was detected on Cu/CeO_2_, proving the pure associative mechanism for the normal Cu/CeO_2_ catalyst. However, for the inverse CeO_2_/Cu catalyst (Fig. 4a), the ratio of generated CO_2_ and H_2_ was 3:1 after CO introduction. This meant that besides the surface hydroxyls, active oxygen atoms created by the dissociation of H_2_O were also involved in the reaction. When H_2_O was injected, H_2_ formation could be observed immediately. These observations matched the features of redox mechanism, assuming that H_2_O dissociated on CeO_2_ oxygen vacancy to generate H_2_ and active surface oxygen atoms. After H_2_O injection, we removed the surface oxygen with H_2_ reduction and preserved the surface hydroxyls^50^, after which CO was purged into the system to conduct the TPSR test again. The subsequent CO treatment gave a CO_2_:H_2_ ratio of 2:1, showing the typical results of associative mechanism. The TPSR experiment was cycled three times in a row, and it gave very repeatable results for both catalysts. Thus, it turned out that both the redox and associative mechanism were present in the WGS reaction catalyzed by the inverse CeO_2_/Cu catalyst.

It has been generally believed that the redox mechanism occurs on metals^46,47^ and the associative mechanism dominates at the metal/oxide interface^50,51^. However, redox pathway has now been found on inverse CeO_2_/Cu catalyst. The improved redox properties of CeO_2_ nanoparticles on inverse CeO_2_/Cu played an important role. Temperature-programmed desorption of CO (CO-TPD) was applied to detect the CO sorption on the inverse catalyst. As illustrated in Fig. 4c, after CO pre-adsorption, CO_2_ was the only desorption species on CeO_2_/Cu, showing easy transformation from CO to CO_2_. For better study over CO adsorption, diffuse reflectance infrared Fourier transform spectroscopy (DRIFTS) was introduced. As shown in Fig. 4d, the in situ DRIFTS of the inverse CeO_2_/Cu catalyst under WGS conditions exhibited CO_2_ signal^52^ (2334, 2364 cm^−1^) at the beginning, which indicated the ongoing of WGS reaction. Meanwhile, no signal of CO was detected during the measurement, while normal Cu/CeO_2_ gave CO–Cu^0^ adsorption (2094 cm^−1^)^53,54^ (Supplementary Fig. 10b). Pure Cu showed no adsorption behavior under both WGS and CO modes, suggesting CO adsorption on bulk Cu was not favored (Supplementary Fig. 10c and d). Besides, the results of in situ DRIFTS for CO adsorption on the CeO_2_/Cu also only present CO_2_ signal, which showed that active surface species of the catalyst was reduced by CO (Supplementary Fig. 10a). Besides, we measured the reaction orders of CO and H_2_O for different catalysts. As shown in Supplementary Fig. 11, the H_2_O reaction order increased when CeO_2_ content was elevated, giving the catalyst stronger capability to consume H_2_O. The CO reaction order on inverse CeO_2_/Cu was 0.75, which suggested CO was comparatively insufficient during WGS reaction. All the above data demonstrated the easy transformation from CO to CO_2_ on inverse CeO_2_/Cu, reflecting unique redox properties of the CeO_2_ nanoparticles. The surface oxygen of inverse CeO_2_/Cu was proved to be flexible under CO, which facilitated the formation of defect sites.

### Role of the surface defects in the catalysts

**Fig. 5:**
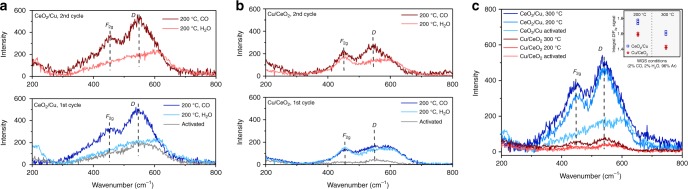
Examination of defect sites in the catalysts. **a** In situ Raman spectra of the inverse CeO_2_/Cu with CO/H_2_O switch under 200 °C. **b** In situ Raman spectra of the normal Cu/CeO_2_ with CO/H_2_O switch under 200 °C. **c** In situ Raman under the WGS conditions for both catalysts, the inset figure gave *D*/*F*_2g_ integral ratio at different temperatures

The formation of defect sites on inverse CeO_2_/Cu were evidenced by in situ Raman measurements, since ex situ Raman and XPS results did not show pronounced Ce^3+^ signals. As shown in Fig. 5a, the Raman spectra of the inverse CeO_2_/Cu after H_2_ activation gave a hump at 600 cm^−1^, which was ascribed to the intrinsic defects^55,56^. When CO was filled in, surface oxygen was removed, forming two characteristic peaks located at 546 and 456 cm^−1^, respectively. The peak centered at 546 cm^−1^ (*D* peak) resulted from defects where Ce^4+^ was replaced by Ce^3+^ ^56^, and the peak centered at 456 cm^−1^ was typical vibration mode (*F*_2g_) of fluorite-type structure^56,57^. It is surprising that the Raman signals of the inverse CeO_2_/Cu showed that the defect *D* peak was even more pronounced than *F*_2g_ peak. When H_2_O was introduced, the intensity of the *D* peaks decreased apparently, which meant the Ce^3+^ defects were filled. The second cycle gave the same results. The introduction of CO induced the creation of surface defects through the reaction:

$${\mathrm{CO + O}} \to {\mathrm{CO}}_{\mathrm{2}}$$

$${\mathrm{CO + OH}} \to {\mathrm{CO}}_{\mathrm{2}}{\mathrm{ + 1/2H}}_{\mathrm{2}}$$

For the normal Cu/CeO_2_ catalyst (Fig. 5b), the intensity of Ce^3+^ *D* peaks was clearly weakened. The generation and elimination of defects with the CO/H_2_O switch was also sluggish. In situ Raman under WGS conditions (Fig. 5c) showed consistent results. The inverse CeO_2_/Cu also gave more pronounced signal of surface defects under the WGS conditions, with *D*/*F*_2g_ integral ratio of 1.8. These results were totally different from those of pure CeO_2_ sample (Supplementary Fig. 12), which gave very strong *F*_2g_ peak and nearly no defect peak. The in situ Raman results have confirmed the enrichment of Ce^3+^ defect sites in inverse CeO_2_/Cu under WGS conditions, which further proved the enhanced redox properties of CeO_2_ on inverse CeO_2_/Cu.

For both catalysts, metallic Cu was the only phase detected under the WGS conditions. Supplementary Fig. 13 displayed the TPSR results of inverse CeO_2_/Cu and normal Cu/CeO_2_ catalysts. The WGS reaction began to occur at ~100 °C. After TPSR, the following H_2_-TPR measurement gave no H_2_ reduction peaks, which suggested Cu remained in fully metallic state during the WGS reaction. This finding was in accordance with the former work reported by Barrio et al.^29^ However, considering the very high WGS activity of the inverse CeO_2_/Cu catalyst, the active site was more likely to locate at Cu–CeO_2_ interfaces rather than metallic Cu.

## Discussion

The mixed oxides of Cu–Zn–Al have been applied as industrial WGS catalysts for decades. Though lots of efforts have been made, few reports have found Cu-based catalysts as effective as Cu–Zn–Al, especially under industrial atmosphere. Herein, the inverse CeO_2_/Cu catalyst showed greatly promoted WGS activity, which was five times higher than that of normal Cu/CeO_2_ catalyst. The WGS conversion of the inverse CeO_2_/Cu was very close to that of commercial Cu–Zn–Al under industrial WGS atmosphere, approaching the equilibrium maximum. The high WGS activity for the inverse CeO_2_/Cu catalyst originated from its unique structure, where bulk–nano interfaces were constructed. When Cu was loaded on CeO_2_, sintering was inevitable as the aggregation of Cu species lowered the surface energy. The dynamic elimination of Cu–CeO_2_ interfaces caused rapid deactivation. Thus, for normal Cu/CeO_2_ catalyst, though Cu–CeO_2_ interfaces were created, the elimination due to structural change would take place even in H_2_ pre-treatment. This deactivation resulted in very low WGS *r* in activity and stability tests (Fig. 1a, d). Considering its high Cu dispersion, the turnover frequency (TOF) of normal Cu/CeO_2_ was calculated for single site on Cu–CeO_2_ interface (see detail in Supplementary Methods). The derived TOF was 0.056 s^−1^, as shown in Table 1.

**Fig. 6:**
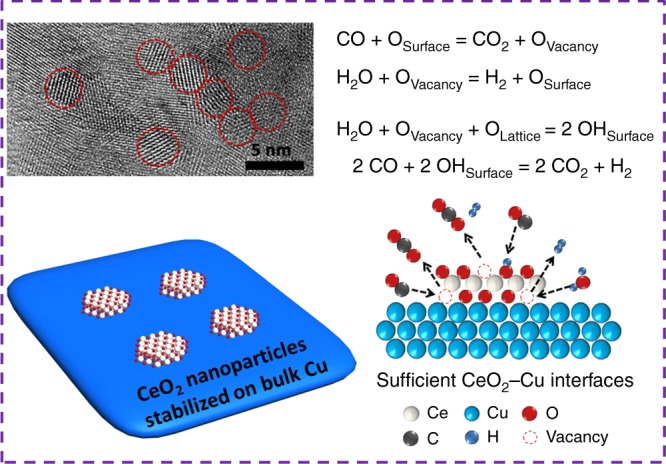
Role of inverse CeO_2_/Cu in catalyzing the WGS reaction. Role of the CeO_2_–Cu interfaces in catalyzing the WGS reaction on inverse CeO_2_/Cu catalyst

Meanwhile, for inverse CeO_2_/Cu catalyst, bulk Cu formed steady structure under WGS conditions. Small CeO_2_ nanoparticles were dispersed on bulk Cu and were also very stable against sintering. The maximized and stabilized bulk–nano interfaces in the inverse CeO_2_/Cu catalyst gave significant WGS promotion. The high WGS activity resulted from the structural nature of the inverse CeO_2_/Cu catalyst, which lied in creation and preservation of stabilized bulk–nano interfaces. A theoretical model has been built to calculate TOF for the inverse CeO_2_/Cu. A 3 nm CeO_2_ nanoparticle with 231 Ce atoms was loaded on metallic Cu. As shown in Supplementary Fig. 14, 16 Ce atoms were located at the periphery of Cu–CeO_2_ interface. Based on this model, the TOF of the inverse CeO_2_/Cu catalyst for single site on CeO_2_–Cu interface was 0.058 s^−1^ (see detail in Supplementary Methods). The similar TOF of both catalysts proved that the Cu–CeO_2_ and CeO_2_–Cu interfaces possessed the same intrinsic activities. As shown in Table 1, the amount of interface sites for inverse CeO_2_/Cu was four times higher than that for normal Cu/CeO_2_. The highly promoted WGS activity of inverse CeO_2_/Cu originated from the enrichment of interface sites. Besides, owing to the strong Cu–CeO_2_ interaction, the redox properties of inverse CeO_2_/Cu catalyst were improved. The surface oxygen of inverse CeO_2_/Cu catalyst was proved to be flexible under CO, leading to the formation of defects. H_2_O dissociated at the defects, promoting the WGS activity via a combination of both the associative mechanism and redox mechanism. The discussion of reactivity and mechanism was concluded in Fig. 6.

The pursuing of highly active and stable catalysts would never be out of date. A strategy to fabricate robust WGS catalyst was proposed, where the key was the construction of bulk–nano interfaces. By using aerosol-spray method, we developed the CeO_2_/Cu catalysts with inverse configuration. Small CeO_2_ nanoparticles (2–3 nm) were stabilized on bulk Cu, forming stable CeO_2_–Cu interfaces under reaction conditions. The enrichment and preservation of such interfaces resulted in significant promotion in activity. The inverse CeO_2_/Cu catalyst exhibited great WGS activity, which was at least five times higher than other reported Cu catalysts. The improved redox properties of the inverse CeO_2_/Cu catalyst facilitated the H_2_O dissociation and CO oxidation, boosting WGS activity via the combination of associative and redox mechanism. Catalyst with sufficient bulk–nano interfaces has now been proved to give excellent WGS performances under realistic conditions, and show great potentials in practical applications and other catalytic systems.

## Methods

### Catalyst preparation

In a typical synthesis of Cu_*a*_Ce_*b*_O_*x*_, 4 mmol of metal nitrates (99%, Tianjin Kermal Factory) were added to 60 ml of absolute ethanol (99%, Tianjin Fuyu Fine Chemical Reagent Factory). This mixture was stirred for 10 min and then put into a ultrasonic humidifier (30 W, 1.5 MHz). Industrial N_2_ served as the carrier gas to bring the spray generated through sonication into the tube furnace (pre-heated to 400 °C). The mist drop evaporated and the precursor underwent decomposition and self-assembly. The metal oxides products were spherical nanoparticles, which were collected on a piece of filter paper and dried overnight at 60 °C. Then, the obtained powder was calcined in air for 4 h (1 °C min^−1^ of ramping rate). The obtained catalysts were nominated as Cu_*a*_Ce_*b*_O_*x*_, of which *a* and *b* referred to the molar ratio of corresponding element, respectively. Commercial Cu–Zn–Al catalyst (37 wt% CuO, 52 wt% ZnO, 11 wt% Al_2_O_3_, determined by EDS) was bought from Sichuan Shutai Chemical Engineering Company.

### Transmission electron microscope (TEM)

All images of TEM were taken on a JEOL JEM-2100F microscope, of which the acceleration voltage was 200 kV. The images of HR-TEM were obtained by using a Philips Tecnai F20 instrument with the acceleration voltage of 200 kV. The element mapping results and EDS analysis were acquired from the same machine under STEM mode.

### X-ray diffraction (XRD)

For the XRD data, all experiments were performed on a PANalytical B.V. X’pert3 powder diffractometer with CuK_α_ radiation (*λ* = 0.15418 nm). Accelerating voltage and current of 40 kV and 40 mA were applied for ex situ and in situ modes. Ex situ XRD patterns were obtained by using a PIXcell^D^ detector in the 2*θ* range of 20‒80°. For the in situ XRD experiments, an Anton Paar XRK900 in situ chamber was applied. The XRD data were measured from 50 to 400 °C in 5% H_2_/Ar mixture (30 cm^3^ min^−1^).

### X-ray photoelectron spectroscopy (XPS)

The XPS measurements were performed on an Axis Ultra XPS spectrometer from Kratos, Japan. The operation was under 225 W of accelerating voltage and Al K_α_ radiation. The C 1*s* line located at 284.8 eV was used to calibrate each spectra for accurate binding energies.

### Raman spectroscopy

Ex situ and in situ Raman spectra were obtained by excitation of the catalysts at 633 nm laser, using a LabRAM HR800 Raman spectrometer (Horiba Jobin Yvon) with the range from 200 to 800 cm^−1^ in a spectral resolution of 2 cm^−1^.

### Temperature-programmed surface reaction (TPSR)

An online mass spectrometer (Ametek LC-D200M) was used to analyze the outlet gases for TPSR, as well as CO-TPD. 100 mg of each sample were treated with 5% H_2_/Ar (30 cm^3^ min^−1^) for 30 min at 300 °C before test. The sample was then flushed under pure He for 1 h. For CO-TPD, the samples were purged with 5% CO/Ar (30 cm^3^ min^−1^) for 0.5 h, and then flushed under pure He for another 1 h at room temperature. Afterwards, the samples were heated from room temperature to 500 °C under He flow. For TPSR, the catalysts were heated from 120 to 400 °C under 2%H_2_O, 2% CO/Ar gas flow (30 cm^3^ min^−1^). For the examination of reaction mechanisms, the samples went through WGS reaction for 1 h at 200 °C after H_2_ reduction. The catalysts were then purged by Ar at 200 °C, followed by the switch of 2%CO/Ar, pure Ar, 2%H_2_O/Ar, pure Ar, 5%H_2_/Ar, pure Ar, and 2%CO/Ar to collect the mass spectrometer signal. The heating rate for all tests was set as 10 °C min^−1^.

### Catalytic tests and kinetics measurement

A fixed-bed reactor with diameter of 1 cm was used for the WGS reaction tests. In order to give accurate results, a thermocouple connected with a PID temperature controller was mounted on top of the catalyst bed. The catalyst powder (100 mg) was loaded and reduced with 5% H_2_/Ar mixture for 0.5 h at 300 °C. The reaction gas contained 2% CO and 10% H_2_O, balanced with N_2_. The total gas hourly space velocity (GHSV) was 42,000 cm^3^ g^−1^ h^−1^. In order to prevent water condensation, all pipes of the reactor were binded with heating belts. The activity results of catalysts were measured from 150 to 400 °C as 50 °C per step. Each catalyst was tested repeatedly to rule out the uncertainty. A Gasboard 3500 IR spectroscopy (WuhanSifang Company, China) was used to analyze all the outlet gases online. For the stability tests, the CO conversion data of the catalysts were continuously recorded under 250 °C for 50 h. The mass of the catalysts and the flow rate of reaction gas were tuned to keep the CO conversion under 15%. The WGS activity was measured by means of CO conversion, which was defined as the following formula:

1 $$X_{{\mathrm{CO}}}\left( {\mathrm{\% }} \right) = \left( {n_{{\mathrm{CO}}}^{{\mathrm{in}}} - n_{{\mathrm{CO}}}^{{\mathrm{out}}}} \right)/n_{{\mathrm{CO}}}^{{\mathrm{in}}}\times100{\mathrm{\% }}$$

For the kinetic tests, 20 mg of catalysts were pre-reduced with 5% H_2_/Ar mixture. The apparent activation energy (*E*_a_) of each catalyst for WGS reaction was obtained by keeping 10% equal CO conversion with the regulation of reaction temperature, catalysts mass, and stream flow rate. The calculation of reaction rate (*r*) for WGS followed the equation:

2 $$r = F \times {\mathrm{CO}}_{{\mathrm{converted}}}/W$$

where *F* is the total flow rate of the reaction stream (mol s^−1^). *r* is the WGS reaction rate by means of CO (mol g^−1^ s^−1^). CO_converted_ is the concentration of converted CO on the IR spectroscopy and *W* is the mass of the catalyst (g).

The reaction orders of CO and H_2_O for the catalysts were measured under 250 °C. The WGS activity was recorded while the concentration of CO or H_2_O in the reaction gas was varied on purpose.

## Supplementary information

Supplementary Information

Peer Review

## Data Availability

The main data supporting the findings of this study are available within the article and its Supplementary information. Extra data are available from the corresponding author upon request.

## References

[1] Kowalczyk Z (1997). Effect of potassium on the kinetics of ammonia synthesis and decomposition over fused iron catalyst at atmospheric pressure. J. Catal..

[2] Koryabkina NA (2003). Determination of kinetic parameters for the water–gas-shift reaction on copper catalysts under realistic conditions for fuel cell applications. J. Catal..

[3] Qiao B (2011). Single-atom catalysis of CO oxidation using Pt1/FeO*x*. Nat. Chem..

[4] Yang M (2014). Catalytically active Au–O(OH)x-species stabilized by alkali ions on zeolites and mesoporous oxides. Science.

[5] Guo LW (2016). Contributions of distinct gold species to catalytic reactivity for carbon monoxide oxidation. Nat. Commun..

[6] Yao S (2017). Atomic-layered Au clusters on α-MoC as catalysts for the low-temperature water–gas shift reaction. Science.

[7] Liu L, Corma A (2018). Metal catalysts for heterogeneous catalysis: from single atoms to nanoclusters and nanoparticles. Chem. Rev..

[8] Campbell CT (2002). The effect of size-dependent nanoparticle energetics on catalyst sintering. Science.

[9] Carter JH (2017). Activation and deactivation of gold/ceria-zirconia in the low-temperature water–gas-shift reaction. Angew. Chem. Int. Ed..

[10] Dai Y, Lu P, Cao Z, Campbell CT, Xia Y (2018). The physical chemistry and materials science behind sinter-resistant catalysts. Chem. Soc. Rev..

[11] Ratnasamy T (2009). Water gas shift catalysis. Catal. Rev. Sci. Eng..

[12] Gawande MB (2016). Cu and Cu-based nanoparticles: synthesis and applications in catalysis. Chem. Rev..

[13] Campbell CT, Daube KA (1987). A surface science investigation of the water–gas-shift reaction on Cu (111). J. Catal..

[14] Nakamura J, Campbell JM, Campbell CT (1990). Kinetics and mechanism of the water-gas shift reaction catalysed by the clean and Cs-promoted Cu (110) surface: a comparison with Cu (111). J. Chem. Soc. Faraday Trans..

[15] Li Y, Fu Q, Flytzani-Stephanopoulos M (2000). Low-temperature water-gas shift reaction over Cu- and Ni-loaded cerium oxide catalysts. Appl. Catal. B.

[16] Qi X, Flytzani-Stephanopoulos M (2004). Activity and stability of Cu–CeO2 catalysts in high-temperature water–gas shift for fuel-cell applications. Ind. Eng. Chem. Res..

[17] Zerva C, Philippopoulos CJ (2006). Ceria catalysts for water gas shift reaction: influence of preparation method on their activity. Appl. Catal. B.

[18] Mudiyanselage K (2013). Importance of the metal–oxide interface in catalysis: in situ studies of the water–gas shift reaction by ambient-pressure x-ray photoelectron spectroscopy. Angew. Chem. Int. Ed..

[19] Aranifard S, Ammal SC, Heyden A (2014). On the importance of metal–oxide interface sites for the water–gas shift reaction over Pt/CeO2 catalysts. J. Catal..

[20] Xu M (2018). Insights into interfacial synergistic catalysis over Ni@TiO2-x catalyst toward water-gas shift reaction. J. Am. Chem. Soc..

[21] Rodriguez JA, Hrbek J (2010). Inverse oxide/metal catalysts: a versatile approach for activity tests and mechanistic studies. Surf. Sci..

[22] Senanayake SD (2013). Unique properties of ceria nanoparticles supported on metals: novel inverse ceria/copper catalysts for CO oxidation and the water-gas shift reaction. Acc. Chem. Res..

[23] Rodriguez JA (2016). Inverse oxide/metal catalysts in fundamental studies and practical applications: a perspective of recent developments. J. Phys. Chem. Lett..

[24] Rodriguez JA, Ma S, Liu P, Hrbek J, Evans J, Pérez M (2007). Activity of CeOx and TiOx nanoparticles grown on Au(111) in the water–gas shift reaction. Science.

[25] Shi J (2014). Nanoporous gold-supported ceria for the water–gas shift reaction: UHV inspired design for applied catalysis. Catal. J. Phy. Chem. C.

[26] Shi J (2016). A versatile sol–gel coating for mixed oxides on nanoporous gold and their application in the water gas shift reaction. Catal. Sci. Technol..

[27] Shi J (2017). Steam reforming of methanol over oxide decorated nanoporous gold catalysts: a combined in situ FTIR and flow reactor study. Phys. Chem. Chem. Phys..

[28] Rodriguez JA (2009). Water-gas shift reaction on a highly active inverse CeOx/Cu (111) catalyst: unique role of ceria nanoparticles. Angew. Chem. Int. Ed..

[29] Barrio L (2010). Unraveling the active site in copper-ceria system for the water-gas shift reaction: in situ characterization of an inverse powder CeO2-x/CuO–Cu catalyst. J. Phy. Chem. C.

[30] Lu. Y (1999). Aerosol-assisted self-assembly of mesostructured spherical nanoparticles. Nature.

[31] Jin Z, Xiao M, Bao Z, Wang P, Wang J (2012). A general approach to mesoporous metal oxide microspheres loaded with noble metal nanoparticles. Angew. Chem. Int. Ed..

[32] Yan H (2016). Promoted multimetal oxide catalysts for the generation of hydrogen via ammonia decomposition. J. Phy. Chem. C.

[33] Gawade P, Mirkelamoglu B, Ozkan US (2010). The role of support morphology and impregnation medium on the water gas shift activity of ceria-supported copper catalysts. J. Phy. Chem. C.

[34] Si R, Zhang L, Chan SW, Flytzani-Stephanopoulos M (2012). Structure sensitivity of the low-temperature water–gas shift reaction on Cu–CeO2 catalysts. Catal. Today.

[35] Saw ET (2014). Bimetallic Ni–Cu catalyst support on CeO2 for high-temperature water–gas shift reaction: methane suppression via enhanced CO adsorption. J. Catal..

[36] Miao D, Goldbach A, Xu H (2016). Platinum/apatite water-gas shift catalysts. ACS Catal..

[37] Fu Q, Saltsburg H, Flytzani-Stephanopoulos M (2003). Active nonmetallic Au and Pt species on ceria-based water–gas shift catalysts. Science.

[38] Yan H, Qin XT, Yin Y, Teng YF, Jin Z, Jia CJ (2018). Promoted Cu–Fe3O4 catalysts for low-temperature water gas shift reaction: optimization of Cu content. Appl. Catal. B.

[39] Yang F (2011). CeO2↔CuOx interactions and the controlled assembly of CeO2 (111) and CeO2 (100) nanoparticles on an oxidized Cu (111) substrate. J. Phys. Chem. C.

[40] Konsolakis M (2016). The role of copper–ceria interactions in catalysis science: recent theoretical and experimental advances. Appl. Catal. B.

[41] Wang X (2018). Sacrificial adsorbate strategy achieved strong metal-support interaction of stable Cu nanocatalysts. ACS Appl. Energy Mater..

[42] Fu J, Ji W, Shen Z, Tang S (1999). Preparation and characterization of CuO nanocrystals. J. Solid State Chem..

[43] Reina TR (2016). The role of Au, Cu & CeO2 and their interactions for an enhanced WGS performance. Appl. Catal. B.

[44] Wang W (2017). Crystal plane effect of ceria on supported copper oxide cluster catalyst for CO oxidation: importance of metal-support interaction. ACS Catal..

[45] Yahiro H (2007). Study on the supported Cu-based catalysts for the low-temperature water–gas shift reaction. Catal. Today.

[46] Gokhale AA, Dumesic JA, Mavrikakis M (2008). On the mechanism of low-temperature water gas shift reaction on copper. J. Am. Chem. Soc..

[47] Lin CH, Chen CL, Wang JH (2011). Mechanistic studies of water–gas-shift reaction on transition metals. J. Phy. Chem. C.

[48] Kalamaras CM, Americanou S, Efstathiou AM (2011). “Redox” vs. “associative formate with –OH group regeneration” WGS reaction mechanism on Pt/CeO2: effect of platinum particle size. J. Catal..

[49] Zhai Y (2010). Alkali-stabilized Pt-OHx species catalyze low-temperature water–gas shift reactions. Science.

[50] Fu X (2019). Direct identification of active surface species for the water–gas shift reaction on a gold−ceria catalyst. J. Am. Chem. Soc..

[51] Aranifard S, Ammal SC, Heyden A (2014). On the importance of metal–oxide interface sites for the water–gas shift reaction over Pt/CeO2 catalysts. J. Catal..

[52] Chen CS (2009). Active sites on Cu–SiO2 prepared using the atomic layer technique for a low-temperature water–gas shift reaction. J. Catal..

[53] Chen CS, Lai TW, Chen CC (2010). Effect of active sites for a wate–gas shift reaction on Cu nanoparticles. J. Catal..

[54] Yao SY (2014). Morphological effect of the nanostructured ceria support on the activity and stability of CuO/CeO2 catalysts for water-gas shift reaction. Phys. Chem. Chem. Phys..

[55] Mcbride JR (1994). Raman and x-ray studies of Ce1−xRExO2−y, where RE=La, Pr, Nd, Eu, Gd, and Tb. J. Appl. Phys..

[56] Wu Z (2010). Probing defect sites on CeO2 nanocrystals with well-defined surface planes by Raman spectroscopy and O2 adsorption. Langmuir.

[57] Guo M (2011). UV and visible Raman studies of oxygen vacancies in rare-earth-doped ceria. Langmuir.

