# Supplementary material for: Construction of stabilized bulk-nano interfaces for highly promoted inverse CeO2/Cu catalyst
Source: Nat Commun. 2019 Aug 2;10:3470. doi: 10.1038/s41467-019-11407-2 (PMC6677889; doi:10.1038/s41467-019-11407-2)
Supplement: Supplementary file 1 — Supplementary Information [file 41467_2019_11407_MOESM1_ESM.pdf]

Supplementary information for:

Construction of stabilized bulk-nano interfaces for highly promoted inverse CeO_2/Cu catalyst

Han Yan[1], *Chun Yang*[1], *Wei-Peng Shao*[1], *Li-Hua Cai*[1], *Wei-Wei Wang*[1], *Zhao Jin*[1] *& Chun-Jiang Jia*[1]*

[1]Key Laboratory for Colloid and Interface Chemistry, Key Laboratory of Special Aggregated Materials, School of Chemistry and Chemical Engineering, Shandong University, Jinan 250100, China.

*Correspondence to: jiacj@sdu.edu.cn.

Table of Contents

Supplementary Methods

Supplementary Figures

Supplementary Tables

Supplementary References

Supplementary Methods:

Surface area of catalysts. The nitrogen sorption measurements were operated on a Builder SSA-4200 surface area analyzer at 77 K. The samples went through a degassing process under vacuum at 200 °C for 6 h. Based on the Brunauer-Emmett-Teller (BET) method, the specific surface area of each sample was calculated.

Temperature program reduction by hydrogen (H_2-TPR). A Builder PCSA-1000 adsorption instrument was used for H_2-TPR. 30 mg of the catalysts were sieved and loaded in the quartz reactor. Each sample was heated in pure O_2 at 300 °C for 0.5 h before measurement. With the ramping rate of 10 °C min^{-1}, the TPR tests were carried out by reducing the catalysts with 5 % H_2/Ar mixture from room temperature to 500 °C.

***In-situ* DRIFTS**. A BrukerVertex 70 spectrometer was used to accomplish the *in-situ* DRIFTS measurements. The spectrometer was equipped with a mercury-cadmium-telluride (MCT) detector and liquid nitrogen was used as the cooling reagent. Also, the diffuse reflectance *in-situ* cell (Harrick system) with CaF_2 windows was essential. The sample powder (ca. 20 mg) was treated with 5% H_2/Ar for 0.5 h at 300 °C as the activation process. For WGS conditions, after cooling down to 200 °C under N_2 (30 cm^3 min^{-1}), the background spectrum was collected via 32 scans at 4 cm^{-1} resolution. The reaction gas consisted of 2%CO/2%H_2O/96%N_2, and was introduced into the *in-situ* chamber (30 cm^3 min^{-1}) after the collection of background spectrum. Continuous recording of the IR profiles was maintained for 0.5 h to acquire equilibrium results. As for CO adsorption tests, the data were collected every minute when 2%CO/98%N_2 and pure N_2 were used as adsorption and flush gas, respectively. All analysis of DRIFTS results was accomplished by using OPUS software.

Ultraviolet-visible (UV-vis) spectroscopy. The UV-vis spectra were recorded on a UV/Vis spectrophotometer (Evolution 220, ISA-220 accessory, Thermo Scientific) using a built-in 10 mm silicon photodiode with a 60 mm Spectralon sphere.

Theoretical model of CeO_2 nanoparticle on Cu. By using the Materials Visualizer of Materials Studio, CeO_2 clusters with the size of 24.5 Å x 24.5 Å x 16.4 Å were placed on the Cu(111) of 7.67 nm x 7.67 nm,. The interface between CeO_2 clusters and Cu was (00-1) plane. The upper surface was (001) plane. The front and back sides were (010) and (0-10) plane, respectively. The left and right sides were (-100) and (100) plane, respectively. The CeO_2 clusters consisted of 6 layers, and each layer contained 41, 40, 41, 40, 37 and 32 Ce atoms, respectively (from bottom to top). There were 231 Ce atoms in total. The exposed facets after cutting the 4 corners were (111), (1-11), (1-11) and (-1-11), respectively.

Cu dispersion and Cu surface area. The Cu dispersion (D) was measured by performing selective N_2O oxidation at 50 °C. The catalysts first underwent H_2-TPR process described above until 400 °C. Then the samples were cooled to 50 °C and purged with helium. The metallic Cu atoms at the surface were selectively oxidized in a 20% N_2O/N_2 gas mixture (30 cm^3 min^{-1}) for 0.5 h. After that, the catalysts were flushed with He again and cooled to room temperature, followed by launching another H_2-TPR run to 400 °C. The hydrogen consumption was obtained through integral of peak area. The calculation of D was described as follows.

All copper atoms went through total reduction in the first TPR run:

$$\mathrm{CuO} + \mathrm{H_2} = \mathrm{Cu} + \mathrm{H_2O}, \text{ hydrogen consumption} = X \quad (1)$$

Cu_2O at surface oxidized by N_2O from metallic copper atoms should be reduced in the second TPR run:

$$\mathrm{Cu_2O + H_2 = Cu + H_2O}, \text{ hydrogen consumption } = Y \quad (2)$$

The Cu dispersion (D) was derived as:

$$D = (2 \times Y/X) \times 100\% \quad (3)$$

$$S_{\mathrm{cu}} = D \times N_{\mathrm{av}} \times X_{\mathrm{Cu}} \,/\, (A_{\mathrm{Cu}} \times 1.4 \times 10^{19}) \quad (4)$$

where N_{av} was Avogadro's constant. X_{Cu} represent the Cu metal content. A_{Cu} was the atomic weight of Cu.

Calculation of TOF on Cu-CeO_2 and CeO_2-Cu interface. For the inverse CeO_2/Cu catalyst, we calculated the TOF value for a single site on the CeO_2-Cu interface with the modified formula[1]:

$$\mathrm{TOF} = \frac{r * N_{\mathrm{av}} * m_{\mathrm{CeO_2}}}{X_{\mathrm{CeO_2}}} \frac{1}{16\pi d} \quad (5)$$

Where r was the reaction rate, N_{av} was Avogardro's constant, m_{CeO_2} was the weight of the CeO_2 nanoparticle (assuming the semispherical shape, derived from its volume and density, 7.21 g cm^{-3}), X_{CeO_2} was the CeO_2 concentration of the catalyst, 16 was the site density of Ce atoms along the periphery, and d was the crystalline size of CeO_2 (3 nm). The calculation process has been reported.[1]

For the normal Cu/CeO_2 catalyst, we applied the same formula: [1]

$$\mathrm{TOF} = \frac{r * N_{\mathrm{av}} * m_{\mathrm{Cu}}}{X_{\mathrm{Cu}}} \frac{1}{8\pi d} \quad (6)$$

Where r was the reaction rate, N_{av} was Avogardro's constant, m_{Cu} was the weight of the Cu nanoparticle (assuming the semispherical shape, derived from its volume and density, 8.92 g cm^{-3}), X_{Cu} was the Cu concentration of the catalyst, 8 was the site density of Cu atoms along the periphery, calculated by π/2d(Cu-O), where d(Cu-O) was the length of Cu-O bond (0.195 nm), and d was the crystalline size of Cu ($d \approx 1/D$, 1.3 nm). The calculation process has also been reported. [1]

The amounts of interface sites (A) could be calculated with the formula:

$$A = \frac{r * N_{\mathrm{av}}}{\mathrm{TOF}} \quad (7)$$

Where r was the reaction rate and N_{av} was Avogardro's constant.

Supplementary Figures:

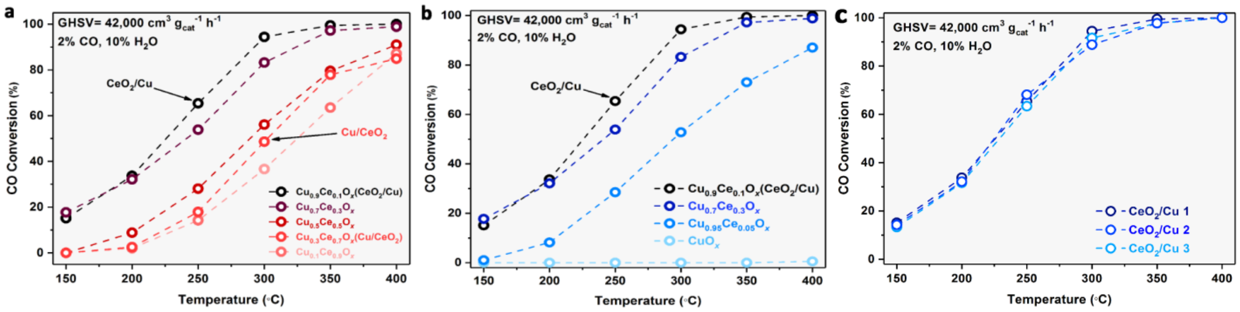

Supplementary Fig. 1 Water-gas shift (WGS) activities of the catalysts prepared by aerosol-spray method. (**a**) Temperature-dependent activity of WGS reaction on all catalysts, $Cu_{0.9}Ce_{0.1}O_x$ was nominated as CeO_2/Cu, $Cu_{0.3}Ce_{0.7}O_x$ was nominated as Cu/CeO_2; (**b**) Temperature-dependent activity of WGS reaction on Cu-riched catalysts; **(c)** Repeated evaluation of WGS activities on inverse CeO_2/Cu. (1atm total pressure, 100 mg of catalysts, 2% CO, 10% H_2O and the rest of N_2, GHSV = 42,000 cm^3 g^{-1} h^{-1})

Supplementary Fig. 1a showed the WGS activities of all prepared catalysts. With Cu content elevating, the WGS conversion also increased. The inverse CeO_2/Cu catalyst with Cu/Ce molar ratio of 9:1 exhibited the best activity. Further increase of Cu/Ce ratio to 9.5:0.5 led to less active catalyst, as shown in Supplementary Fig. 1b. Every catalyst was tested at least twice to rule out the uncertainty. The inverse CeO_2/Cu was chosen as an example. The tested catalysts were separately prepared before each measurement. As shown in Supplementary Fig. 1c, the WGS activities were nearly the same.

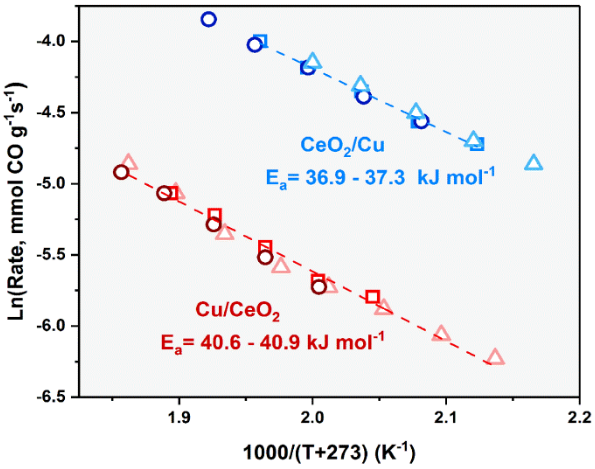

Supplementary Fig. 2 Kinetics of the catalysts. Arrhenius plots of WGS reaction on the inverse CeO_2/Cu catalyst and normal Cu/CeO_2 catalyst.

Supplementary Fig. 2 displayed the Arrhenius plots of inverse and normal catalysts. Repeated measurements showed that WGS on inverse CeO_2/Cu gave an apparent energy (E_a) of 36.9 – 37.3 kJ mol^{-1}, which was about 4 kJ mol^{-1} lower than that (40.6 – 40.9 kJ mol^{-1}) found on normal Cu/CeO_2.

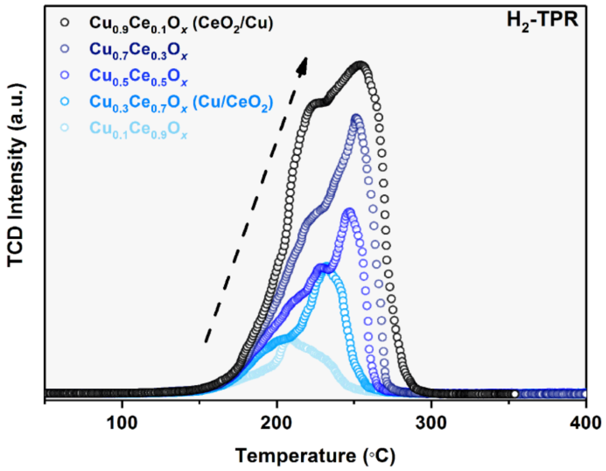

Supplementary Fig. 3 Redox properties of the catalysts. Profiles of the temperature-programmed reduction of H_2 (H_2-TPR) for all the Cu-CeO_2 catalysts.

Supplementary Fig. 3 gave the results of H_2-TPR. The H_2 consumption increased with the elevation of Cu content. Furthermore, the starting temperature of reduction also shifted to lower region when Cu content increased, which suggested that the redox properties for the catalysts were improved.

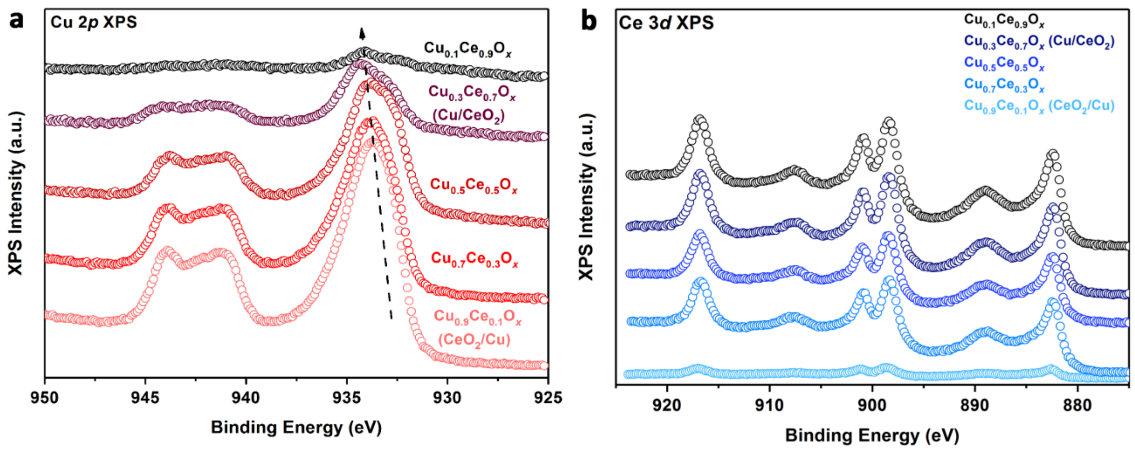

Supplementary Fig. 4 Chemical States of Cu and Ce in the catalysts. (**a**) Cu 2*p* X-ray photoelectron spectroscopy (XPS) spectra of the fresh catalysts; (**b**) Ce 3*d* XPS spectra of the fresh catalysts.

Supplementary Fig. 4 showed XPS results of fresh catalysts. The Cu 2*p* spectra in Supplementary Fig. 4a gave a shift to higher binding energy when CeO_2 content was elevated, indicating electron transfer from CuO to CeO_2. This shift evidenced Cu-CeO_2 interaction, and corresponded well to H_2-TPR results in Supplementary Fig. 3. When CeO_2 content elevated, more electrons transferred from CuO to CeO_2, which added the difficulty of CuO reduction. The Ce 3*d* spectra in Supplementary Fig. 4b gave only Ce^{4+} peaks, which demonstrated CeO_2 was not incorporated into CuO lattice.

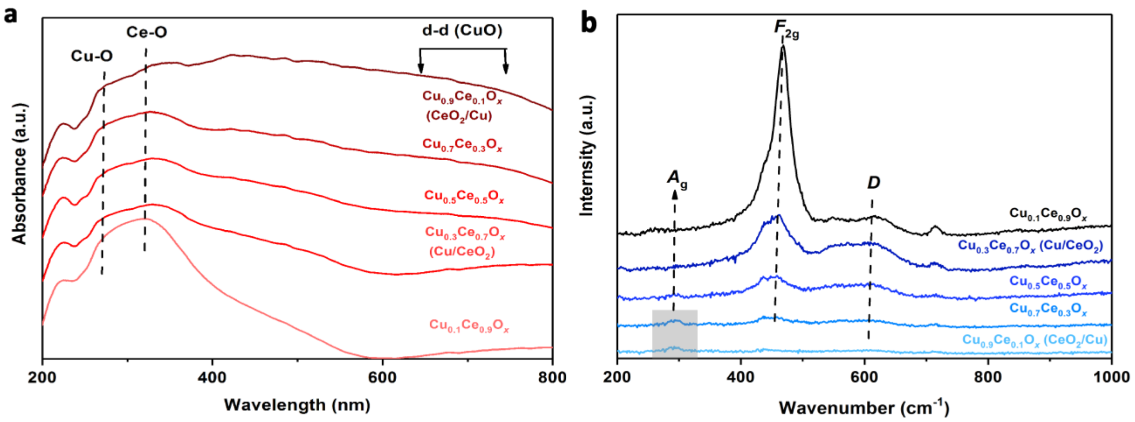

Supplementary Fig. 5 Spectroscopy evidences of Cu-CeO_2 interaction. (**a**) Ultraviolet-visible (UV-vis) spectra over the fresh catalysts; (**b**) Raman spectra over the fresh catalysts.

Supplementary Fig. 5 exhibited UV-vis and Raman spectra. The UV-vis spectra of the inverse CeO_2-Cu (Supplementary Fig. 5a) showed shifting and broadening of the adsorption edge, convincing this Cu-Ce interaction. In Supplementary Fig. 5b, the fresh inverse CeO_2-Cu catalyst only showed a weak peak of Raman mode A_g, which belonged to cupric oxide.

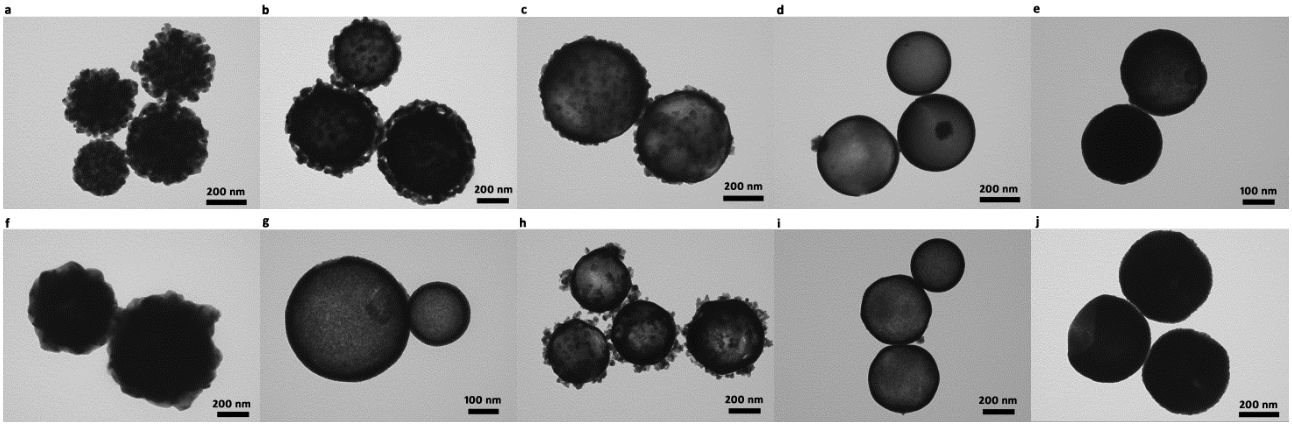

Supplementary Fig. 6 Transmission electron microscope (TEM) observations of the catalysts. TEM images of fresh (**a**-**e**) and used (**f**-**j**) catalysts. (**a**, **f**) $Cu_{0.9}Ce_{0.1}O_x$(CeO_2/Cu); (**b**, **g**) $Cu_{0.7}Ce_{0.3}O_x$; (**c**, **h**) $Cu_{0.5}Ce_{0.5}O_x$; (**d**, **i**) $Cu_{0.3}Ce_{0.7}O_x$ (Cu/CeO_2); (**e**, **j**) $Cu_{0.1}Ce_{0.9}O_x$.

Supplementary Fig. 6 displayed TEM images of the fresh and used catalysts. The fresh catalyst gave morphology of microspheres with size from 200 nm to 500 nm. After WGS reaction, the microspheres aggregated and formed bulk particles. With CeO_2 content elevating, the spherical morphology was better preserved.

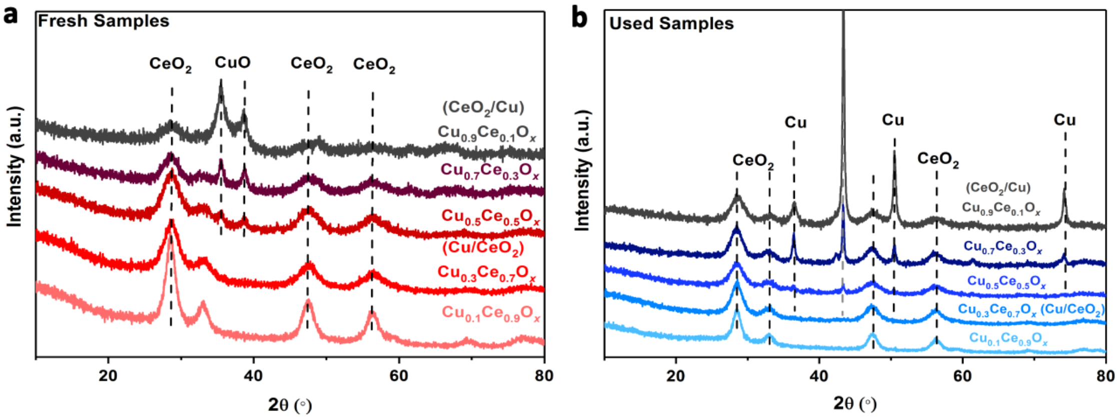

Supplementary Fig. 7 Structural and phase evolution of the catalysts during the WGS reaction. X-ray diffraction (XRD) patterns of (**a**) fresh catalysts and (**b**) catalysts after temperature-dependent catalytic tests (used catalysts).

Supplementary Fig. 7 exhibited the XRD results of fresh and used catalysts. For the catalysts with Cu/Ce ratio of 1:9 and 3:7, no CuO peaks could be observed, indicating high dispersion of Cu species. After WGS reaction, catalysts with Cu/Ce ratio of 9:1 and 7:3 gave sharp metallic Cu peaks, which suggested sintering of catalysts took place.

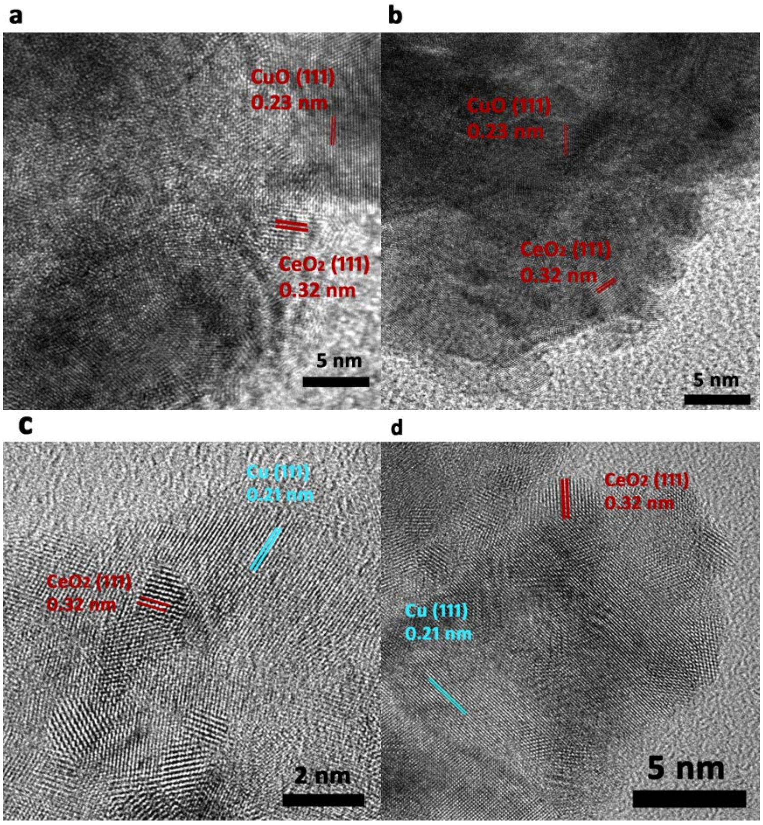

Supplementary Fig. 8 Dispersion of CeO_2 nanoparticles on the Cu species. (**a, b**) High resolution TEM (HR-TEM) images of the fresh inverse CeO_2/Cu catalyst; (**c, d**) HR-TEM images of the used inverse CeO_2/Cu catalyst.

Supplementary Fig. 8 displayed the HR-TEM images for fresh and used catalysts. Supplementary Fig. 8a and b showed interplanar spacing of 0.23 and 0.32 nm, corresponding to lattice fringes of CuO (111) and CeO_2 (111), respectively. Supplementary Fig. 8c and d showed interplanar spacing of 0.21 and 0.32 nm, corresponding to lattice fringes of Cu (111) and CeO_2 (111), respectively.

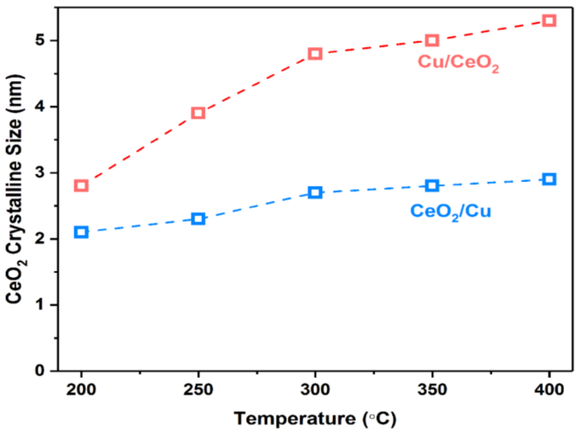

Supplementary Fig. 9 Variation of CeO_2 crystalline sizes during *in-situ* XRD tests. Varying trend of CeO_2 crystalline size for different catalysts in the *in-situ* XRD tests (5% H_2-Ar, calculated with Scherrer formula).

Supplementary Fig. 9 showed the variation of CeO_2 crystalline size for CeO_2/Cu and Cu/CeO_2 catalysts in the *in-situ* XRD experiments. The signals before 200 °C were not detectable. For CeO_2/Cu, the average size of CeO_2 nanoparticles was 2.1 nm at 200 °C, and held up to 2.9 nm at 400 °C. For Cu/CeO_2, CeO_2 nanoparticles grew from 2.8 to 5.3 nm. The CeO_2/Cu catalyst possessed more stable CeO_2-Cu interfaces with stabilization of CeO_2 on bulk Cu.

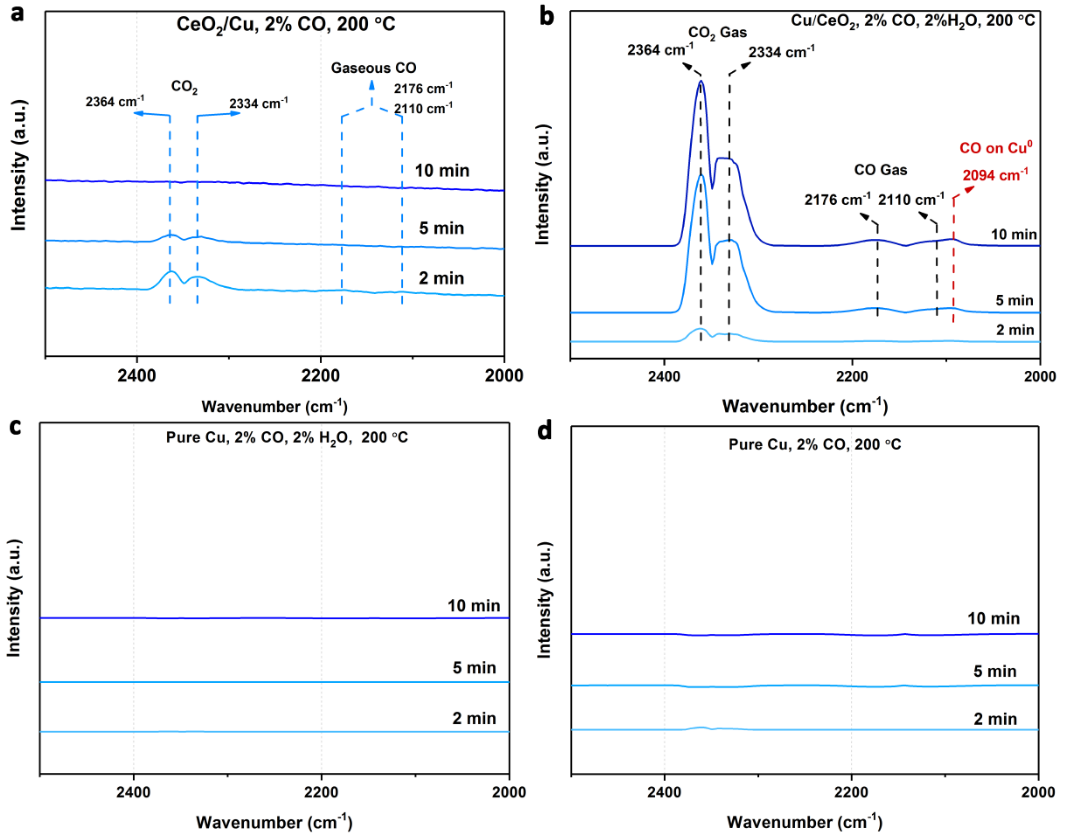

Supplementary Fig. 10 CO adsorption on different catalysts. *In-situ* diffuse reflectance infrared Fourier spectroscopy (DRIFTS) results of (**a**) inverse CeO_2/Cu; (**b**) normal Cu/CeO_2; (**c**) and (**d**) pure Cu sample at 200 °C.

All samples were pre-reduced at 300 °C for 0.5 h and measured for DRIFTS. As shown in Supplementary Fig. 10a, the CO adsorption DRIFTS gave gaseous CO and CO_2 signals for inverse CeO_2/Cu. For bulk Cu (Supplementary Fig. 10c and d), there were no sorption behavior. We believed that bulk Cu had hardly any CO adsorption ability, which was also evidenced by the data in Supplementary Fig. 1b, showing pure CuO was totally inactive for WGS reaction. Normal Cu/CeO_2 showed weak CO adsorption on metallic Cu (2094 cm^{-1}, Supplementary Fig. 10b), indicating Cu was kept at metallic state under reaction conditions.

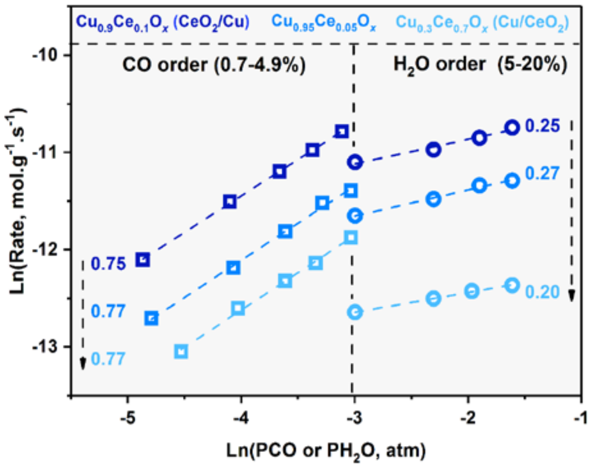

Supplementary Fig. 11 Reaction orders of reactants over different catalysts. Power law with the effects of CO and H_2O on reaction rate over the inverse CeO_2/Cu, normal Cu/CeO_2 and $Cu_{0.95}Ce_{0.05}O_x$ catalysts.

The CO and H_2O reaction orders were measured and the corresponding data were present in Supplementary Fig. 11. The CO reaction orders for CeO_2/Cu and Cu/CeO_2 catalysts were similar (0.75–0.77). The H_2O reaction orders of the catalysts were much lower, averaging between 0.20–0.27. The low H_2O orders demonstrated the strong ability of consuming H_2O on the catalysts. The higher CO orders (0.75–0.77) suggested that CO was comparatively insufficient during WGS reaction. Besides, the H_2O reaction order decreased with increasing CeO_2 amount, which suggested that CeO_2 was responsible for H_2O dissociation.

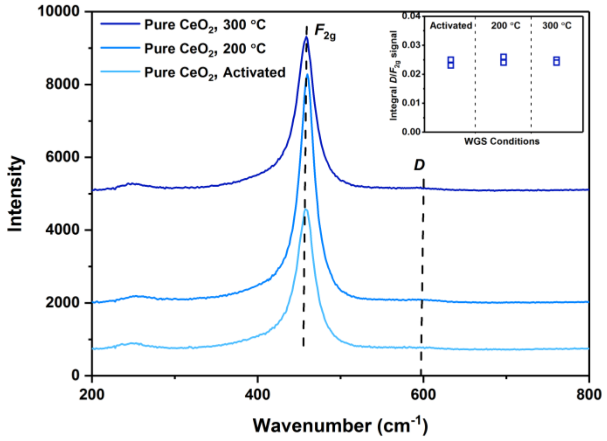

Supplementary Fig. 12 Raman spectra of the CeO_2. *In-situ* Raman results of pure CeO_2 with excitation laser of 633 nm, the inset figure gave D/F_{2g} integral ratio at different temperatures.

As shown in Supplementary Fig. 12, pure CeO_2 gave very strong Raman intensity under *in-situ* mode. The F_{2g} peak was pronounced under different temperatures, and the D/F_{2g} integral ratio was very low (0.02–0.03). The unique Raman spectra for inverse CeO_2/Cu resulted from its dark color. When 633 nm of excitation laser was implied, most of the incident ray was absorbed and signals from the surface were amplified.[2]

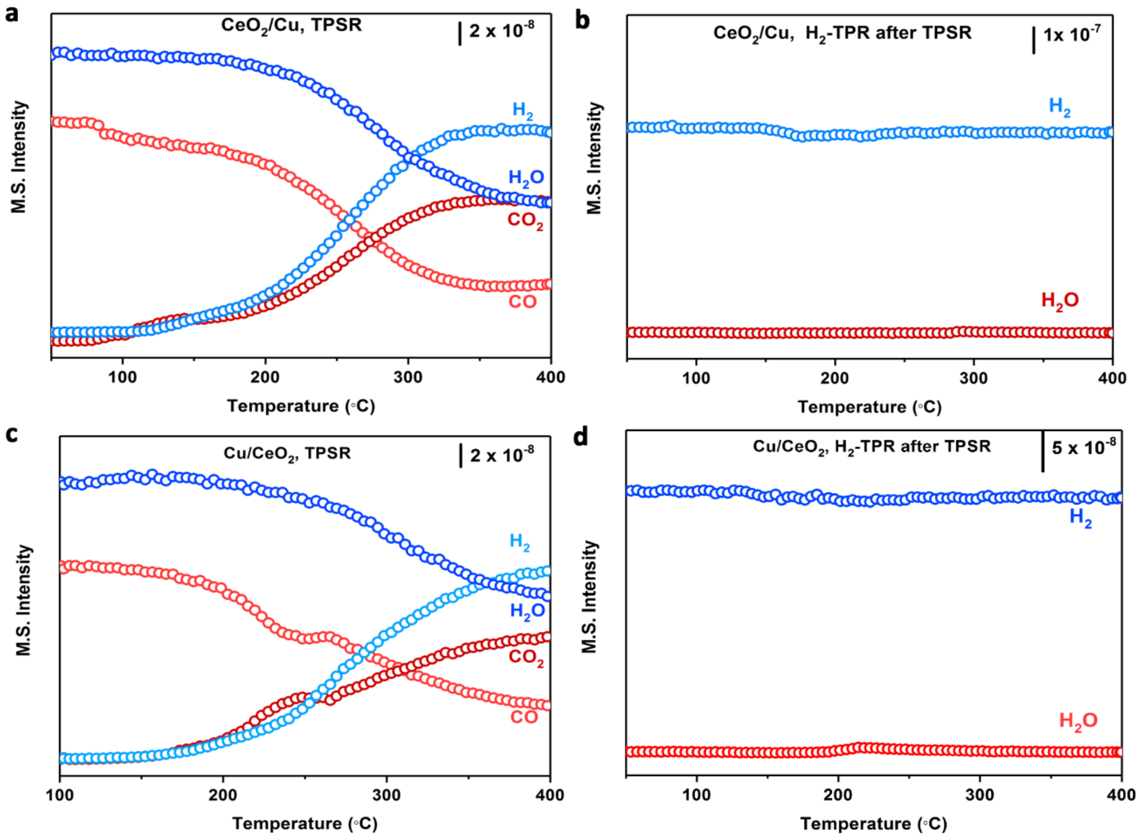

Supplementary Fig. 13 Exclusion of Cu oxides during the WGS reaction. (**a**) Temperature-programmed surface reaction (TPSR) results of inverse CeO_2/Cu; (**b**) H_2-TPR profiles of inverse CeO_2/Cu after TPSR; (**c**) TPSR results of normal Cu/CeO_2; (**d**) H_2-TPR profiles of normal Cu/CeO_2 after TPSR. (1atm total pressure, 100 mg of catalysts, 2% CO, 2% H_2O and the rest of N_2, total flow = 30 cm^3 min^{-1})

Supplementary Fig. 13 exhibited the results of TPSR for CeO_2/Cu and Cu/CeO_2 catalysts. Both samples showed WGS activity with generation of CO_2 and H_2. After TPSR, the samples were held in inert gas and reduced with H_2. The H_2-TPR gave no reduction peak, which suggested that Cu species in both catalysts kept metallic Cu^0 state during WGS reaction.

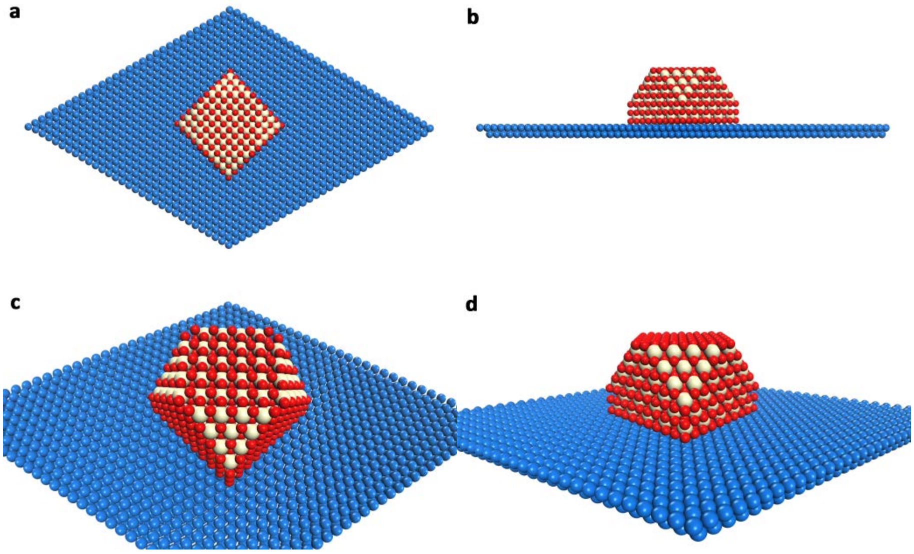

Supplementary Fig. 14 Model of the inverse CeO_2/Cu interface. A 3 nm CeO_2 nanoparticle with 231 Ce atoms was loaded on metallic Cu, 16 Ce atoms was located at the periphery of Cu-CeO_2 interfaces. (**a**) Top view; (**b**) Side view; (**c**) and (**d**) Front view.

Supplementary Tables:

Supplementary Table 1 Physicochemical properties of fresh and used catalysts.

**Catalyst**	**Cu wt.% [a]**		d_{CeO_2} **/nm [b]**		d_{Cu}**/nm [b]**		S_{BET}**/m^2 g^{-1}**		**Cu dispersion [c]**
Pure Cu	87.5[d]	98.6[e]	-[d]	-[e]	19.9[d]	108.1[e]	41.3[d]	8.6[e]	0.1%
CeO_2/Cu ($Cu_{0.9}Ce_{0.1}O_x$)	61.5	82.9	2.6	2.7	8.0	101.2	47.7	16.2	9.1%
$Cu_{0.7}Ce_{0.3}O_x$	41.5	54.1	3.2	3.9	16.6	23.0	45.8	28.5	54.7%
$Cu_{0.5}Ce_{0.5}O_x$	25.3	31.6	3.4	4.5	12.2	19.8	48.5	37.1	72.5%
Cu/CeO_2 ($Cu_{0.3}Ce_{0.7}O_x$)	10.7	13.4	3.6	4.7	-	-	46.5	42.8	79.6%
$Cu_{0.1}Ce_{0.9}O_x$	2.8	3.9	5.6	6.6	-	-	52.8	50.9	84.6%

[a] Weight ratio determined by EDS.

[b] CuO for fresh samples, Cu for used samples. Calculated from the XRD patterns with Scherrer formula.

[c] Determined by N_2O chemisorption

[d] Data acquired from fresh catalysts

[e] Data acquired from used catalysts (Total 6 h of on-stream time)

Supplementary Table 2 Comparisons of WGS reaction rates toward the catalysts. (300 °C)

Catalyst	Conditions	E_a/kJ mol^{-1}	Rate/10^{-6} mol g^{-1} s^{-1}	Reference
Inverse CeO_2/Cu	2%CO, 10%H_2O, balance N_2	37	47.5	This work
Normal Cu/CeO_2	2%CO, 10%H_2O, balance N_2	40	10.2	This work
Cu-Ce(La)O_x	2%CO, 10%H_2O, balance He	30	9.0	[3]
CeO_x/Cu	1%CO, 3%H_2O, balance He	—	9.8	[4]
$Cu_{0.3}Fe_{0.7}O_x$	2%CO, 10%H_2O, balance N_2	47	12.2	[5]
5Ni5Cu/CeO_2[a]	7%CO, 22%H_2O, 10% CO_2, 20% H_2, balance He	41	22.1	[6]
Pt-Ca-HAP	15%CO, 30%H_2O, 5% CO_2, 40% H_2, balance N_2	87	21.6	[7]
Pt-Ce(La)O_x	11%CO, 26%H_2O,7% CO_2, 26% H_2, balance He	75	45	[8]

[a] Measured at 350 °C.

Supplementary References:

1. Jia, A., Jiang, S., Lu, J., & Luo, M. Study of Catalytic Activity at the CuO-CeO_2 Interface for CO Oxidation. *J. Phys. Chem. C* **114**, 21605–21610 (2010).

2. Guo, M. et al. UV and Visible Raman Studied of Oxygen Vacancies in Rare-Earth-Doped Ceria. *Langmuir* **27**, 3872–3877 (2011).

3. Li, Y., Fu, Q. & Flytzani-Stephanopoulos, M. Low-temperature water-gas shift reaction over Cu- and Ni-loaded cerium oxide catalysts. *Appl. Catal. B* **27**, 179–191 (2000).

4. Barrio, L, *et al.* Unraveling the active site in copper-ceria system for the water-gas shift reaction: *in situ* characterization of an inverse powder CeO_{2-x}/CuO-Cu catalyst. *J. Phy. Chem. C* **114**, 3580–3587 (2010).

5. Yan, H., Qin, X. T., Yin, Y., Teng, Y. F., Jin, Z. & Jia, C. J. Promoted Cu-Fe_3O_4 catalysts for low-temperature water gas shift reaction: optimization of Cu content. *Appl. Catal. B* **226**, 182–193 (2018).

6. Saw, E. T. et al. Bimetallic Ni-Cu catalyst support on CeO_2 for high-temperature water-gas shift reaction: methane suppression via enhanced CO adsorption. *J. Catal*. **314**, 32–46 (2014).

7. Miao, D., Goldbach, A. & Xu, H. Platinum/apatite water-gas shift catalysts. *ACS Catal*. **6**, 775–783 (2016).

8. Fu, Q., Saltsburg, H. & Flytzani-Stephanopoulos, M. Active nonmetallic Au and Pt species on ceria-based water-gas shift catalysts. *Science* **301**, 935–938 (2003).
