# Supplementary material for: Construction of stabilized bulk-nano interfaces for highly promoted inverse CeO2/Cu catalyst
Source: Nat Commun. 2019 Aug 2;10:3470. doi: 10.1038/s41467-019-11407-2 (PMC6677889; doi:10.1038/s41467-019-11407-2)
Supplement: Supplementary file 2 — Peer Review [file 41467_2019_11407_MOESM2_ESM.pdf]

Reviewers' comments:

Reviewer #1 (Remarks to the Author):

The authors present a very interesting synthesis method to prepare inverse ceria/copper catalysts, which are exceptionally active and stable. This part of the work and the characterization is very good. The limited mechanistic studies and analysis is in contrast poor and contradicts the current consensus in the field. The experiments do not support their conclusion about having a redox mechanism dominating the process. As an example, the authors mention to have only observed CO2 in the DRIFT data during the WGS reaction at 200 C but not CO, and that leads to a redox mechanism. It is amply established from surface science and powder catalysts that CO is weakly adsorbed in any form of copper (See the careful DRIFT work from Martinez-Arias et al. that shows that CO is only stable below 150 C on Cu+ sites, and at much lower temperatures on metallic copper and references therein, Phys. Chem. Chem. Phys., 2014, 16, 17183-17195, Morphological effects of the nanostructured ceria support on the activity and stability of CuO/CeO2 catalysts for the water-gas shift reaction). The authors show that reduction of the ceria nanoparticles by CO gives CO2, which is well established due to the reducibility of ceria, but that doesn't mean that the same mechanism happens catalytically during the oxidation of CO by water. In fact, extensive work in the literature details mechanistic studies using a combination of in situ experiments, on high surface area and planar catalysts, and theory which show that the redox mechanism dominates on metals and the more efficient associative mechanism at the metal/oxide interface. For example, ceria alone is a bad catalyst, gold cannot catalyze the reaction, but ceria/gold is an excellent catalyst [see 2016 review from ref 23 in the manuscript related to ceria/gold and ceria/copper and many refs. therein; see also from A. Heyden et al., On the importance of metal–oxide interface sites for the water–gas shift reaction over Pt/CeO2 catalysts. Journal of Catalysis, 2014. 309: p. 314-324, and refs. therein].
Based on the above, I do not recommend publication.

Reviewer #2 (Remarks to the Author):

In this contribution entitled "Construction of stabilized bulk-nano interfaces for highly promoted inverse CeO2/Cu catalyst" by Yan and co-workers the authors describe the preparation of an inverse CeO2/CU catalyst and its application in the water-gas shift reaction. This reaction is of great importance for the future generation of hydrogen and its context in the field of energy harvesting. The topic is timely and the data presented in the paper is interesting and convincing and mostly provides evidence and answers to readers.

However, I have a few problems with the manuscript in its current form. For example the discussion of inverse CeO2/Au (and other oxides) for ambient pressure WGS is missing, see for example:

Shi et al.,, Steam reforming of methanol over oxide decorated nanoporous gold catalysts: a combined in situ FTIR and flow reactor study, Phys. Chem. Chem. Phys. 2017, 19, 8880-8888
or
Shi et al., A versatile sol-gel coating for mixed oxides on nanoporous gold and their application in the water gas shift reaction, Catalysis Science & Technology 2016, 6, 5311-5319.
Or
Shi et al., Nanoporous Gold-Supported Ceria for the Water–Gas Shift Reaction: UHV Inspired Design for Applied Catalysis, J. Phys. Chem. C 2014, 118, 29270-29277.

These publications are quite relevant and the novelty of the manuscript might be reduced. Some further points:

On page 4 (end of introduction): it should read CO oxidation and not reduction

When comparing the inverse catalyst with the supported Cu the composition of both catalysts is very different (10.7 wt% as opposed to 82.9 wt% in the inverse system). How do the different rates of both systems compare in terms of specific surface area and the metal loading. (rates are stated/compared per gram only). The authors claim that the perimeter between the oxide particle and the Cu is responsible but this remains elusive as it could not be quantified for both catalysts.
It appears that the Cu supported on CeO_2 remains oxidized also under WGS and reducing atmosphere. Could this also be an explanation for the lower observed activity? On page 10 the authors state "we have further confirmed that for inverse CeO_2/Cu catalyst, metallic Cu is the only active phase detected for WGS". Shouldn't this hold true for the "normal" catalyst and be an explanation for the reduced activity?
In table 1 on page 6 BET surface areas are stated but it is not clear whether they were measured before or after catalysis. The sintering of the Cu particles in case of the inverse system is severe and should be accounted for. When the authors state "after catalysis" a time on stream should be stated as structural changes might be related to this time.
On page 5 when discussing the (apparent) activation barriers the error should be stated. The finding that the Ea for the "normal" Cu/CeO_2 catalyst was 3.7 kJ/mol lower might well be within error margins.
On page 8 when discussing the number of defects (Ce^{3+}) the authors state that the dominant species was Ce^{4+}. This is unexpected as the higher activity of the inverse system is usually ascribed to a higher fraction of Ce^{3+} as the active site for H_2O dissociation. Did the authors also try methods such as Raman spectroscopy to quantify? When discussing the in-situ experiments (depicted in Figure 4) the authors postulate, however, such defect sites. (see also the above mentioned publications)
In Figure 4, regarding the DRIFTS measurements: It appears that the spectra only contain information from the gas phase (namely CO_2). Also the experimental conditions, i.e. the water content was much lower. I would have expected to see water and as the case may be OH from the oxide surface. What could be an explanation? At current I don't see why the DRIFTS contain any additional information than the formation of CO_2 in the gas phase?

Reviewer #3 (Remarks to the Author):

In this manuscript, the authors report on the preparation of an inverse catalyst consisting of rather large Cu nanoparticles that serve as a support for much smaller CeO_2 nanoparticles. Interestingly, such structures appear to show good activity and stability for the water-gas shift (WGS) reaction. With some small exceptions, the authors appear to have done a relatively thorough job in characterizing both the initial structure of the catalyst as well as the final structure under reaction conditions. (The final structure is very different from the initial structure, due to a very large extent of Cu sintering.). Overall, this concept of using relatively large Cu particles as supports for CeO_2 particles is an interesting one, though I do have a few comments for this manuscript:

Did the authors determine the uncertainties based on replicate experiments for the results in Figure 1? It does not seem clear from the methods section. Showing meaningful uncertainty analysis is especially important for justifying the claim that the inverse CeO_2/Cu catalyst has higher activity at long times than does that commercial WGS catalyst. In general, there seems to be a lack of sufficient error analysis in the manuscript.

My other major comments is that the paper would be significantly improved if the authors could demonstrate that the reaction rate on their inverse system is proportional to some measure of the number of interfacial sites. Based on their characterization results and the cartoon pictured in Figure 1(h), can the authors estimate the interfacial areas for some of the catalysts shown in Supplementary figure 1, and perhaps demonstrate that the reaction rate scales with perimeter around the CeO_2

nanoparticles?

It is a little unclear on page 5 when the authors report an activation energy of 37.1 kJ/mol for the inverse catalyst and that Cu-CeO2 is 3.7 kJ/mol higher. The authors then say Cu-CeO2 is expected to be close to 50-60 kJ/mol. Can they explain this apparent discrepancy?

Is it surprising that the CeO2 shows no chemical shifts as the Cu loading increases in Figure S4? Also, is there some reason that the Ce intensity does not appear to change over most of the range of bulk composition changes?

Can the authors comment on how the Cu surface areas / particle sizes compare between the catalyst reported here, the supported Cu/CeO2 catalyst, and the Cu-Zn-Al catalyst?

Finally, while the results are clearly communicated, there are numerous grammatical errors in the manuscript.

Responses to the reviewers' comments

To Reviewer #1:

Comment: *The authors present a very interesting synthesis method to prepare inverse ceria/copper catalysts, which are exceptionally active and stable. This part of the work and the characterization is very good. The limited mechanistic studies and analysis is in contrast poor and contradicts the current consensus in the field. The experiments do not support their conclusion about having a redox mechanism dominating the process. As an example, the authors mention to have only observed* CO_2 *in the DRIFT data during the WGS reaction at 200 C but not CO, and that leads to a redox mechanism. It is amply established from surface science and powder catalysts that CO is weakly adsorbed in any form of copper (See the careful DRIFT work from Martinez-Arias et al. that shows that CO is only stable below 150 C on Cu+ sites, and at much lower temperatures on metallic copper and references therein, Phys. Chem. Chem. Phys., 2014, 16, 17183-17195, Morphological effects of the nanostructured ceria support on the activity and stability of CuO/CeO2 catalysts for the water-gas shift reaction). The authors show that reduction of the ceria nanoparticles by CO gives CO2, which is well established due to the reducibility of ceria, but that doesn't mean that the same mechanism happens catalytically during the oxidation of CO by water. In fact, extensive work in the literature details mechanistic studies using a combination of in situ experiments, on high surface area and planar catalysts, and theory which show that the redox mechanism dominates on metals and the more efficient associative mechanism at the metal/oxide interface. For example, ceria alone is a bad catalyst, gold cannot catalyze the reaction, but ceria/gold is an excellent catalyst [see 2016 review from ref 23 in the manuscript related to ceria/gold and ceria/copper and many refs. therein; see also from A. Heyden et al., On the importance of metal–oxide interface sites for the water–gas shift reaction over Pt/CeO2 catalysts. Journal of Catalysis, 2014. 309: p. 314-324, and refs. therein].*

Based on the above, I do not recommend publication.

Author Reply: The reviewer's kind comment on our work is highly appreciated. We found that our former mechanism study was incomplete and not very deep. The reviewer makes a great point saying associative mechanism is more efficient at the metal/oxide interface. In fact,

recently, by using *in-situ* DRIFTS and TPSR, our group has also proved that associative mechanism is dominant at the Au-CeO_2 interface (Fu X. et al. *J. Am. Chem. Soc*. 2019, 141, 4613). According to the constructive criticism on the mechanism study, we have carefully read the literatures that the reviewer listed and re-designed our experiments. As mentioned by the reviewer, the DRIFTS signal of CO was not detected under WGS conditions. However, we did the DRIFTS measurements just to examine the CO adsorption, although no adsorbed CO was detected, not for exploring the mechanism. **Here, we have carefully re-designed the TPSR experiments to examine the reaction mechanism. The results proved the inverse CeO_2/Cu catalyst followed a combination of associative mechanism and redox mechanism**. As the reviewer's comment said, associative mechanism should not be ruled out for the inverse catalyst. The detailed results were described as follows:

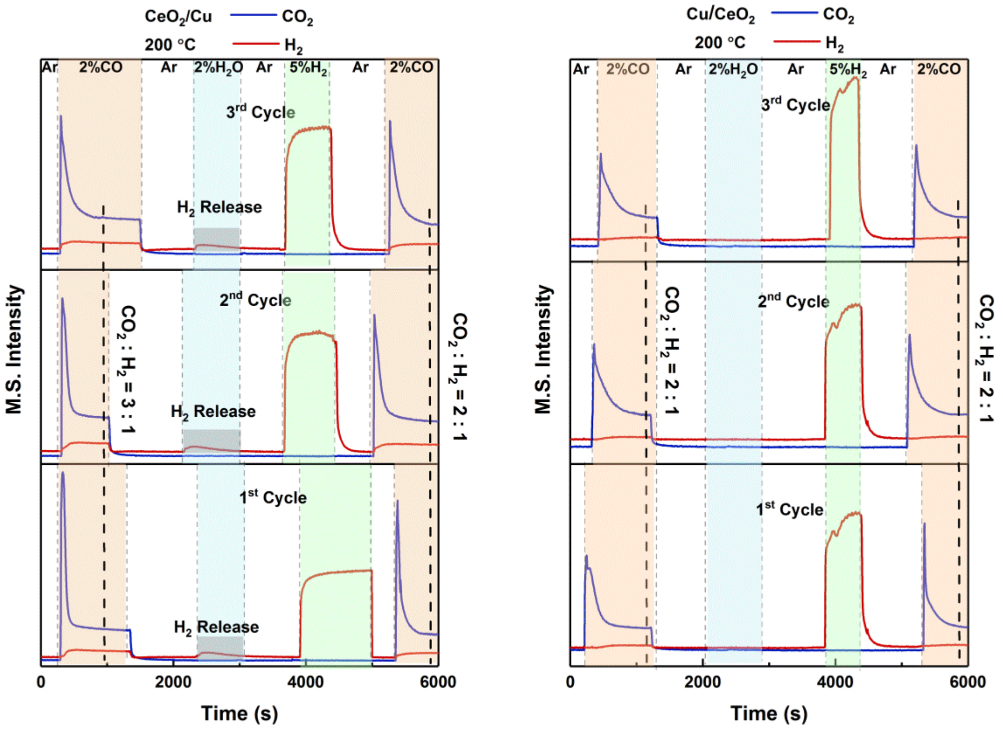

Figure R1 | TPSR on the inverse CeO_2/Cu with consecutive switch of CO, H_2O and H_2 at 200 °C. Stepwise TPSR results of (a) inverse CeO_2/Cu and (b) normal Cu/CeO_2.

Following the associative mechanism, quantified measurement of CO_2 and H_2 concentration in TPSR must give the ratio of 2:1 (Fu X. et al. *J. Am. Chem. Soc*. 2019, 141, 4613), since 2 CO molecules reacted with 2 –OH to generate 2 CO_2 molecules and 1 H_2 (2CO

+ 2–OH → $2CO_2$ + H_2). In the former manuscript, the catalyst was firstly reduced by H_2 and then introduced to H_2O, which was not real WGS condition. Such treatment led to oxidation of Cu by H_2O, causing exorbitant CO_2 : H_2 ratio of 10 : 1, which was much higher than 2 : 1. Therefore, in our original manuscript, combining that H_2 was generated through the disassociation of H_2O on CeO_2/Cu without the presence of CO, we thought the WGS catalyzed by the inverse CeO_2/Cu followed the redox mechanism. Now we have found that such a process was not proper to reflect the real status of the catalyst since the step of H_2O vapor purging caused the over-oxidation of the Cu. **In the revised manuscript, we have modified our TPSR experiments. As shown in Figure R1, the inverse and normal catalysts were firstly treated under WGS conditions, and then purged with Ar, which ensured that the catalysts underwent the real status as the WGS reaction.** Then CO was introduced afterwards to react with the active surface species. At this stage, the normal Cu/CeO_2 gave CO_2 : H_2 ratio of 2 : 1, which corresponded very well to the reaction of 2CO + 2–OH = 2 CO_2 + H_2, proving the pure associative mechanism. After Ar purging for the second time, H_2O was introduced. No H_2 signal was detected by the MS, indicating that the H_2O cannot be converted to H_2 and active oxygen atoms by the normal Cu/CeO_2 catalyst, further proving the associative mechanism. **However, for the inverse CeO_2/Cu catalyst, the CO_2 : H_2 ratio was 3 : 1, not 2 : 1, for the TPSR test.** This meant that besides the surface hydroxyls, active oxygen atoms created by the dissociation of H_2O were also involved in the reaction. Additionally, H_2 was generated after only H_2O injection. These results definitely confirmed that redox mechanism was present for the inverse CeO_2/Cu, together with the associative mechanism. Then, we in purpose used H_2 to remove the surface oxygen but preserved surface hydroxyl on the catalyst, and then CO was filled in again. At this stage, the inverse CeO_2/Cu catalysts gave CO_2 : H_2 ratio of 2 : 1, showing the typical results of associative mechanism, which was due to the consumption of the reactive oxygen species by purged H_2. The same experiment was repeated three times in a row, and the results were very repeatable. **From the above experiments, we confidently concluded that both the redox and associative mechanism were present in the WGS reaction catalyzed by the inverse CeO_2/Cu catalyst. Neither of which could be ruled out. These important results were added in the revised manuscript as Figure 4a and b and the relevant discussion was**

supplemented (please see page 10, line 1–11, page 11, line 1–9).

The dissociation of H_2O to H_2 is directly related to defect sites in the catalyst. To further study the role of defect sites, we designed and performed *in-situ* Raman experiments. The similar procedure with TPSR was applied by using cyclic CO and H_2O treatment under 200 °C. **As shown in Figure R2a,** two characteristic peaks, namely F_{2g} peak of CeO_2[2] and surface defect (oxygen vacancy) peak *of D* (Guo, M. et al. *Langmuir*, 2011, 27, 3872), could be observed. **It is surprising that when CO was introduced, the defect peak (*D*) of inverse CeO_2/Cu was greatly pronounced, even exceeding the CeO_2 F_{2g} peak.** When H_2O was introduced, the defect peak was clearly weakened. This meant that the introduction of CO induced the creation of surface vacancies through the reaction of CO + O → CO_2 and CO + –OH → CO_2 + 1/2H_2. The normal Cu/CeO_2 went through the same experiment (Figure R2b), showing apparently fewer defect sites. These results suggested that for inverse CeO_2/Cu, the surface defect sites related to Ce^{3+} were easily generated under WGS conditions. Also the strong ability of H_2O association catalyzed by the surface defects of CeO_2 ensured the very high WGS activity. **These important data were added in the revised manuscript as Figure 5 and relevant discussion was supplemented (please see page 12, line 3–7, page 13, line 1–10).**

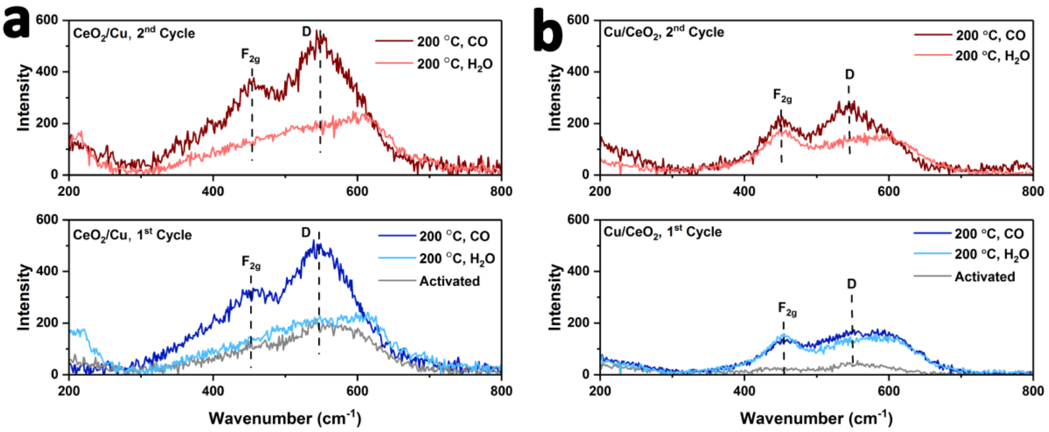

Figure R2 | Defect sites of the catalysts. *In-situ* Raman spectra of (a) inverse CeO_2/Cu and (b) normal Cu/CeO_2 catalysts (633-nm laser).

In-situ Raman experiments were also conducted under the WGS conditions. As shown in Figure R3a, the inverse CeO_2/Cu also gave more pronounced signal of surface defects under the WGS conditions. However, the *in-situ* Raman results of pure CeO_2 gave a dominant CeO_2

F_{2g} peak with very strong intensity (Figure R3b), which was quite different to that for the inverse CeO_2/Cu catalyst. The quantified results showed that the inverse CeO_2/Cu possessed abundant defect sites under WGS conditions, with D/F_{2g} larger than 1.6. These results also suggested that defect sites (Ce^{3+}) were very easily generated under the WGS reaction conditions. We have measured the H_2O reaction orders of the catalysts (Supplementary Figure 11), those were between 0.20–0.27, much lower than those of the CO (0.75–0.77). These results demonstrated the strong ability of consuming H_2O on the inverse catalysts, which was well consistent the above *in-situ* Raman result. **Thus, we concluded that the interface of bulk Cu – nano CeO_2 greatly enhanced the redox properties of the catalyst. These important results were added in the revised manuscript as Figure 5c and relevant discussion was supplemented (please see page 10, line 1–11, page 11, line 1–9).**

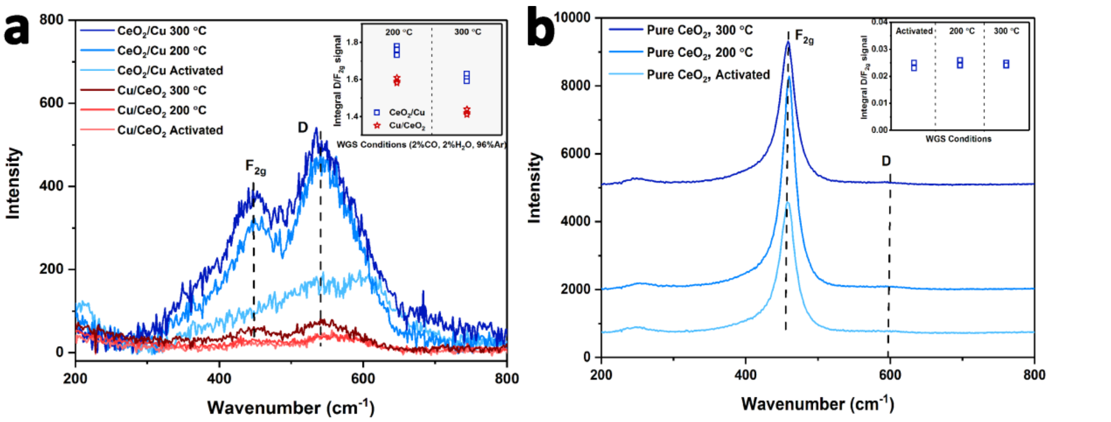

Figure R3 | Defect sites of the catalysts. *In-situ* Raman spectra under WGS conditions of (a) inverse CeO_2/Cu and normal Cu/CeO_2 catalysts and (b) pure CeO_2; the inset Figures give statistical results of the D/F_{2g} integral intensity ratio.

In one word, for the inverse catalyst, the surface oxygen of CeO_2 could be easily removed by CO to form defects. H_2O dissociated on the defects and H_2 was generated, confirming the redox mechanism. Meanwhile, CO also reacted with surface hydroxyl to form CO_2 and H_2, following the associative mechanism. **Therefore, both the redox mechanism and the associative mechanism were present in the WGS reaction catalyzed by the inverse CeO_2/Cu catalyst. The described results were shown as Figure 4 and Figure 5 in the revised manuscript and relevant discussion was supplemented. The cartoon in Scheme 1**

has also been modified.

To Reviewer #2:

Comment 1: *In this contribution entitled "Construction of stabilized bulk-nano interfaces for highly promoted inverse CeO_2/Cu catalyst" by Yan and co-workers the authors describe the preparation of an inverse CeO_2/Cu catalyst and its application in the water-gas shift reaction. This reaction is of great importance for the future generation of hydrogen and its context in the field of energy harvesting. The topic is timely and the data presented in the paper is interesting and convincing and mostly provides evidence and answers to readers.*

Author Reply: We sincerely thank for the reviewer's kind comment.

Comment 2: *However, I have a few problems with the manuscript in its current form. For example the discussion of inverse CeO_2/Au (and other oxides) for ambient pressure WGS is missing, see for example: 1. Shi et al.,, Steam reforming of methanol over oxide decorated nanoporous gold catalysts: a combined in situ FTIR and flow reactor study, Phys. Chem. Chem. Phys. 2017, 19, 8880-8888. 2. Shi et al., A versatile sol-gel coating for mixed oxides on nanoporous gold and their application in the water gas shift reaction, Catalysis Science & Technology 2016, 6, 5311-5319. 3. Shi et al., Nanoporous Gold-Supported Ceria for the Water–Gas Shift Reaction: UHV Inspired Design for Applied Catalysis, J. Phys. Chem. C 2014, 118, 29270-29277. These publications are quite relevant and the novelty of the manuscript might be reduced.*

Author Reply: We greatly thank for the reviewer's comment. These literatures of inverse CeO_2/Au catalysts have given us some inspirations. However, the systematic analyses of catalytic performances, where the activities of inverse catalysts are carefully examined and compared to those of normal catalysts, are still not reported. **As for the novelty of this manuscript, we have mainly focused on synthesizing the practical inverse CeO_2/Cu catalyst with remarkably enhanced WGS activity, which has never been obtained.** Besides, using Cu as catalyst support is comparatively more economic than noble metal. In the revised manuscript, we have cited the articles recommended by the reviewer in the introduction (Reference 25–27). Thanks for the comment again.

Comment 3: *On page 4 (end of introduction): it should read CO oxidation and not reduction*

Author Reply: The reviewer's comment is highly appreciated. We are sorry for this careless mistake. **The revised manuscript has been carefully examined and these errors were corrected**.

Comment 4: *When comparing the inverse catalyst with the supported Cu the composition of both catalysts is very different (10.7 wt% as opposed to 82.9 wt% in the inverse system). How do the different rates of both systems compare in terms of specific surface area and the metal loading. (rates are stated/compared per gram only). The authors claim that the perimeter between the oxide particle and the Cu is responsible but this remains elusive as it could not be quantified for both catalysts.*

Author Reply: We thank for the reviewer's comment. According to the reviewer's concern, we have calculated the Cu surface areas from the dispersion, and the reaction rates in terms of Cu surface area and Cu loading, as shown in Table R1.

Table R1 | Cu content, BET and Cu Surface areas and Cu dispersion.

Catalyst	Cu wt.% [a]		S_{BET}/m^2 g^{-1}		S_{Cu}/m^2 g^{-1}	Cu dispersion[c]
	Fresh	Used	Fresh	Used		
CeO_2/Cu ($Cu_{0.9}Ce_{0.1}O_x$)	61.5	82.9	47.7	16.2	**50.7**	9.1%
Cu/CeO_2 ($Cu_{0.3}Ce_{0.7}O_x$)	10.7	13.4	46.5	42.8	**71.7**	79.6%

As shown in Figure R4a, when the reaction rates were calculated with Cu loading, the normal Cu/CeO_2 gave higher *r* than inverse CeO_2/Cu since it contained much less Cu content. **In our manuscript, we found that the active site for the inverse catalyst lied in the Cu-CeO_2 interface, rather than metallic Cu. With this premise, the WGS reaction rate would not be accurate in terms of Cu loading.** Moreover, the calculated S_{Cu} for inverse CeO_2/Cu and normal Cu/CeO_2 were larger than their S_{BET}. Such phenomenon has been reported in previous literatures (For example, Cu-Zn-Al in Velu S. et al. *Catal. Lett.* 1999, 62, 159). We have

mentioned in the manuscript that CeO_2 was found to participate in N_2O chemisorption, causing higher Cu dispersion (Wang W. et al. *ACS Catal*. 2017, 7, 1313–1329). **We calculated the Cu surface area of Cu-Zn-Al catalyst (19.7 m^2 g^{-1}), which was much lower than that of inverse CeO_2/Cu.** Thus, though the reaction rate of inverse CeO_2/Cu was higher than that of normal Cu/CeO_2 (Figure R4b), the accuracy of the reaction rate derived from S_{Cu} could not be ensured in this system. This was the reason we did not put these data in our manuscript.

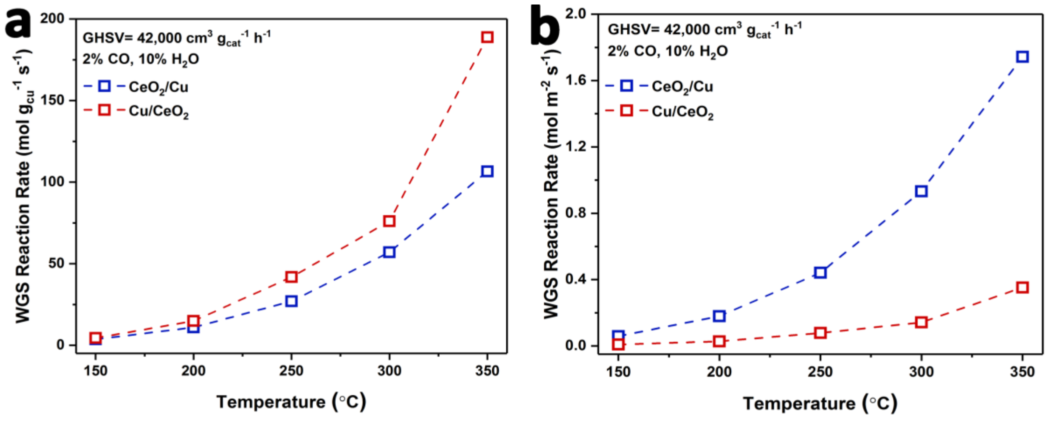

Figure R4 | WGS reaction rates with different calculations. (a) Reaction rates of inverse CeO_2/Cu and normal Cu/CeO_2 in terms of Cu loading; (b) Reaction rates of inverse CeO_2/Cu and normal Cu/CeO_2 in terms of Cu surface area. (1atm total pressure, 100 mg of catalysts, 2% CO, 10% H_2O and the rest of N_2, GHSV = 42,000 cm^3 g^{-1} h^{-1})

As for the reviewer's concern of quantified parameter, it was difficult because we cannot obtain useful information from TEM or STEM. **However, we have made efforts to obtain and compare the TOF between the inverse and normal catalysts, and the results were convincing.** First, we built a model for inverse CeO_2/Cu catalysts (Figure R5), with a CeO_2 nanoparticle (3 nm) on metallic Cu. The CeO_2 nanoparticle consisted of 231 Ce atoms. From this model, we found that there were 16 Ce atoms along the CeO_2-Cu periphery. **Thus, we could calculate the TOF of the single site on the periphery of CeO_2-Cu interface.**

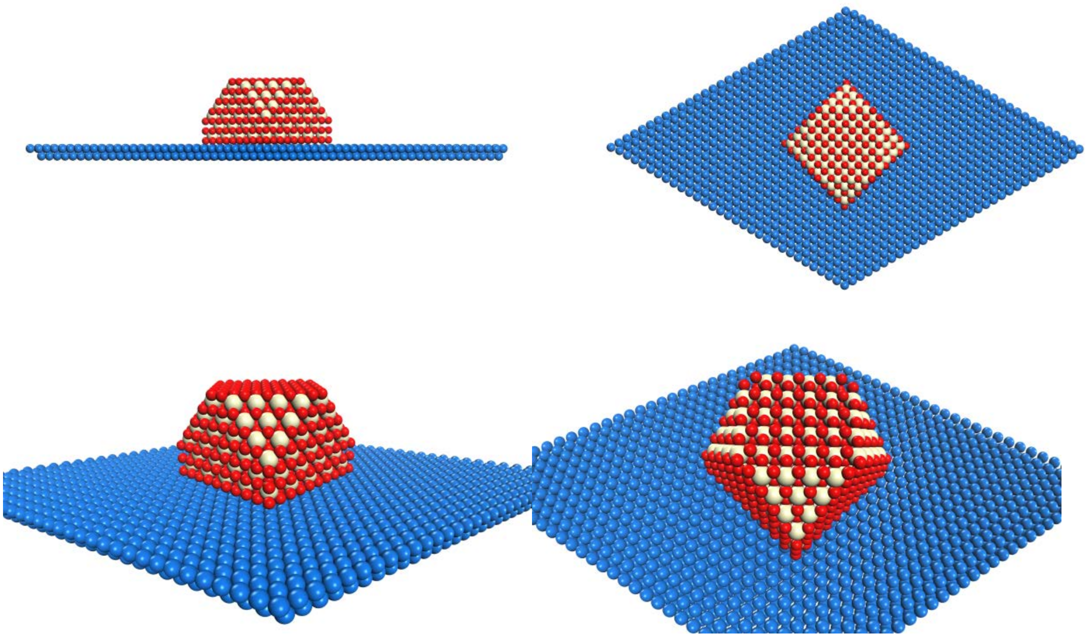

Figure R5 | Model of CeO_2 nanoparticle on Cu metal. The CeO_2 nanoparticle (~3 nm in size) contains 231 Ce atoms, and there are 16 Ce atoms along the Cu-CeO_2 periphery.

Based on this model, we calculated the TOF values of the single site on the periphery of CeO_2-Cu interface with the modified former formula (Jia A.P. et al. *J. Phys. Chem. C*, 2010, 114, 21605) :

$$TOF = \frac{r * \mathrm{N_{av}} * \mathrm{m_{CeO_2}}}{X_{\mathrm{CeO_2}}} \frac{1}{16\pi \mathrm{d}}$$

Where r was the reaction rate, N_{av} was Avogardro's constant, m_{CeO2} was the weight of the CeO_2 nanoparticle (assuming the semispherical shape, derived from its volume and density, 7.21 g cm^{-3}), X_{CeO2} was the CeO_2 concentration of the catalyst, 16 was the site density of Ce atoms along the periphery, and d was the crystalline size of CeO_2 (3 nm). **By using the above formula, the calculated TOF of inverse CeO_2/Cu was 0.058 s^{-1}.**

For the normal Cu/CeO_2 catalyst, we applied the formula below to obtain TOF of the single site on Cu-CeO_2 interface (Jia A.P. et al. *J. Phys. Chem. C*, 2010, 114, 21605):

$$TOF = \frac{r * \mathrm{Nav} * \mathrm{mCu}}{X\mathrm{Cu}} \frac{1}{8\pi \mathrm{d}}$$

Where r was the reaction rate, N_{av} was Avogardro's constant, m_{Cu} was the weight of the Cu nanoparticle (assuming the semispherical shape, derived from its volume and density, 8.92 g cm^{-3}), X_{Cu} was the Cu concentration of the catalyst, 8 was the site density of Cu atoms along the periphery, calculated by π/2d(Cu-O), where d(Cu-O) was the length of Cu-O bond (0.195

nm), and d was the crystalline size of Cu (d ≈ 1/D, 1.3 nm). **By using the above formula, the calculated TOF of normal Cu/CeO_2 was 0.056 s^{-1}.**

The reaction rates (*r*) of the inverse CeO_2/Cu and normal Cu/CeO_2 were 47.3 and 10.2 μmol g^{-1} s^{-1}(Figure 1c). The amounts of interface sites (*A*) could be calculated with the formula:

$$A = \frac{r * \mathrm{Nav}}{TOF}$$

Where *r* was the reaction rate and N_{av} was Avogardro's constant. **The calculated amount of interface sites for inverse CeO_2/Cu was 4.9 × 10^{20}, while that for normal Cu/CeO_2 was 1.1 × 10^{20}.**

The TOF values of the two catalysts were very close, indicating similar nature of the active sites. Besides, the amount of interface sites for inverse CeO_2/Cu was much larger, so we believed the promoted WGS activity of inverse catalyst originated from the enriched CeO_2/Cu interfaces. These important results were present as Supplementary Scheme 1 and in Discussion part of the revised manuscript (please see page 14, line 10–12, line 17–23).

At the same time, we must note that the model for TOF calculations was simplified. The inverse catalyst exhibited more promising redox properties compared with the normal one, which has been shown from the *in-situ* Raman results (Figure R3). Therefore, the difference in activity between the inverse and normal catalyst could also come from the distinct ability in activating the reactants.

Comment 5: *It appears that the Cu supported on CeO_2 remains oxidized also under WGS and reducing atmosphere. Could this also be an explanation for the lower observed activity? On page 10 the authors state "we have further confirmed that for inverse CeO_2/Cu catalyst, metallic Cu is the only active phase detected for WGS". Shouldn't this hold true for the "normal" catalyst and be an explanation for the reduced activity?*

Author Reply: We greatly thank for the reviewer. As pointed by the reviewer, the chemical state of Cu was crucial to the WGS performance of the catalysts. **In our manuscript, we have conducted WGS TPSR for both catalysts, and performed H_2-TPR afterward**

(please see Supplementary Figure 12). There were no reduction peaks after WGS reaction, suggesting Cu remained metallic state for both catalysts.

Comment 6: *In table 1 on page 6 BET surface areas are stated but it is not clear whether they were measured before or after catalysis. The sintering of the Cu particles in case of the inverse system is severe and should be accounted for. When the authors state "after catalysis" a time on stream should be stated as structural changes might be related to this time.*

Author Reply: We appreciate the reviewer's kind comment. The BET surface areas given in Table 1 were measured before catalysis tests. As stressed by the reviewer, the sintering of Cu should definitely be taken into consideration. The BET surface areas of the catalysts after catalysis tests were added in **Table 1 and Supplementary Table 1** of the revised manuscript. The time on stream was of noted in **Supplementary Table 1** of the revised manuscript.

Comment 7: *On page 5 when discussing the (apparent) activation barriers the error should be stated. The finding that the Ea for the "normal" Cu/CeO_2 catalyst was 3.7 kJ/mol lower might well be within error margins.*

Author Reply: The reviewer's comment is highly appreciated. We have repeated the measurement of activation energy toward both catalysts for 3 times. The tested catalysts were separately prepared before each measurement. As shown in Figure R6, the *Ea* for the inverse CeO_2/Cu was from 36.9 to 37.3 kJ/mol, while that for normal Cu/CeO_2 was from 40.6 to 40.9 kJ/mol. **The repeated results suggested that the E_a of inverse CeO_2/Cu was ~4 kJ/mol lower than that of normal Cu/CeO_2. We have replaced Supplementary Figure 2 with Figure R6, and added corresponding discussion of error (please see page 6, line 17–20) in the revised manuscript.**

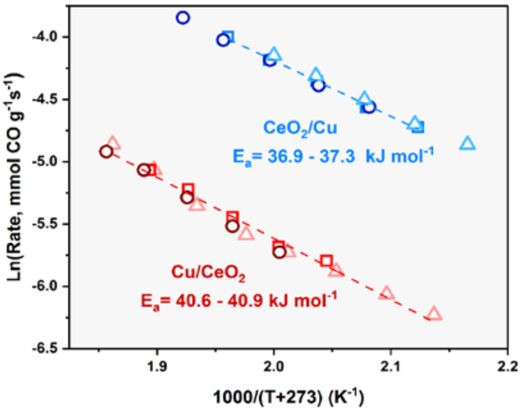

Figure R6 | Replicate measurements of activation energy. Arrhenius plots of WGS reaction on the inverse CeO_2/Cu catalyst and normal Cu/CeO_2 catalyst.

Comment 8: *On page 8 when discussing the number of defects (Ce^{3+}) the authors state that the dominant species was Ce^{4+}. This is unexpected as the higher activity of the inverse system is usually ascribed to a higher fraction of Ce^{3+} as the active site for H_2O dissociation. Did the authors also try methods such as Raman spectroscopy to quantify? When discussing the in-situ experiments (depicted in Figure 4) the authors postulate, however, such defect sites. (see also the above mentioned publications)*

Author Reply: We are grateful to reply the reviewer's comment. ***In-situ* Raman experiments have been performed to check the defect sites.** Firstly we applied cyclic CO and H_2O treatment under 200 °C. **As shown in Figure R2a,** two characteristic peaks, namely F_{2g} peak of CeO_2[2] and surface defect (oxygen vacancy) peak *of D* (Guo, M. et al. *Langmuir*, 2011, 27, 3872), could be observed. **It is surprising that when CO was introduced, the defect peak (*D*) of inverse CeO_2/Cu was greatly pronounced, even exceeding the CeO_2 F_{2g} peak.** When H_2O was introduced, the defect peak was clearly weakened. This meant that the introduction of CO induced the creation of surface vacancies through the reaction of CO + O $\rightarrow$ CO_2 and CO + –OH $\rightarrow$ CO_2 + 1/2H_2. The normal Cu/CeO_2 went through the same experiment (Figure R2b), showing apparently fewer defect sites. These results suggested that for inverse CeO_2/Cu, the surface defect sites related to Ce^{3+} were easily generated under WGS conditions. Also the strong ability of H_2O association catalyzed by the surface defects of CeO_2 ensured the very high WGS activity. **These important data were added in the**

revised manuscript as Figure 5 and relevant discussion was supplemented (please see page 12, line 3–7, page 13, line 1–10).

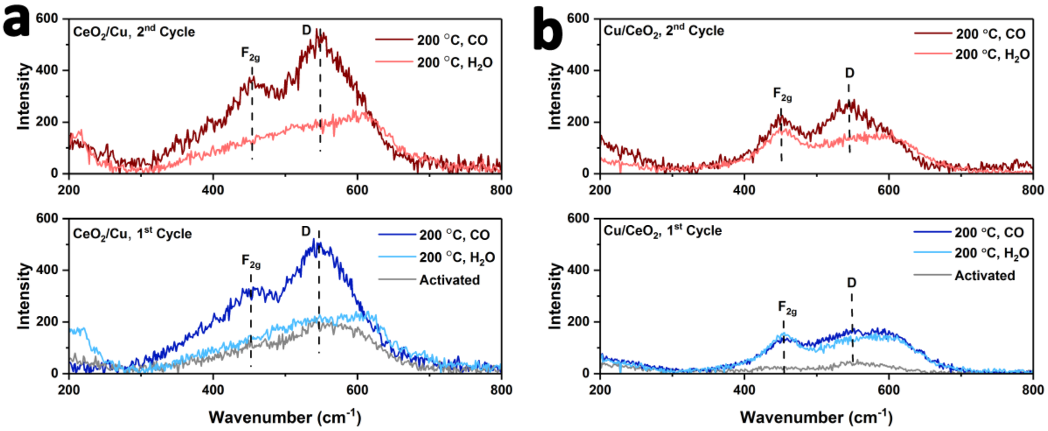

Figure R2 | Defect sites of the catalysts. *In-situ* Raman spectra of (a) inverse CeO_2/Cu and (b) normal Cu/CeO_2 catalysts (633-nm laser).

In-situ Raman experiments were also conducted under the WGS conditions. As shown in Figure R3a, the inverse CeO_2/Cu also gave more pronounced signal of surface defects under the WGS conditions. However, the *in-situ* Raman results of pure CeO_2 gave a dominant CeO_2 F_{2g} peak with very strong intensity (Figure R3b), which was quite different to that for the inverse CeO_2/Cu catalyst. The quantified results showed that the inverse CeO_2/Cu possessed abundant defect sites under WGS conditions, with D/F_{2g} larger than 1.6. These results also suggested that defect sites (Ce^{3+}) were very easily generated under the WGS reaction conditions. We have measured the H_2O reaction orders of the catalysts (Supplementary Figure 11), those were between 0.20–0.27, much lower than those of the CO (0.75–0.77). These results demonstrated the strong ability of consuming H_2O on the inverse catalysts, which was well consistent the above *in-situ* Raman result. **Thus, we concluded that the interface of bulk Cu – nano CeO_2 greatly enhanced the redox properties of the catalyst. These important results were added in the revised manuscript as Figure 5c and relevant discussion was supplemented (please see page 10, line 1–11, page 11, line 1–9).**

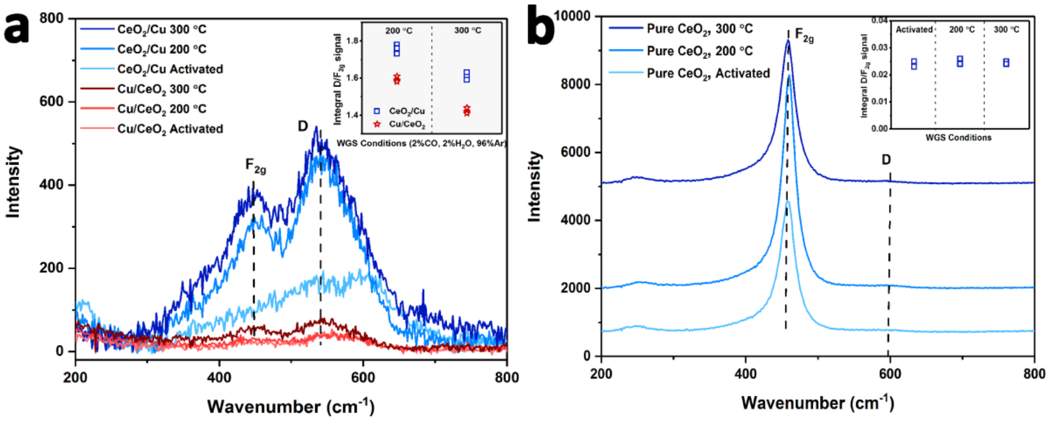

Figure R3 | Defect sites of the catalysts. *In-situ* Raman spectra under WGS conditions of (a) inverse CeO_2/Cu and normal Cu/CeO_2 catalysts and (b) pure CeO_2; the inset Figures give statistical results of the D/F_{2g} integral intensity ratio.

Comment 9: *In Figure 4, regarding the DRIFTS measurements: It appears that the spectra only contain information from the gas phase (namely CO_2). Also the experimental conditions, i.e. the water content was much lower. I would have expected to see water and as the case may be OH from the oxide surface. What could be an explanation? At current I don't see why the DRIFTS contain any additional information than the formation of CO_2 in the gas phase?*

Author Reply: We appreciate the reviewer's constructive comment. The *in-situ* DRIFTS experiments were repeated, and we have also paid attention to the OH region (3600–3800 cm^{-1}). **As shown in Figure R7, terminal and bridged OH could be observed for inverse CeO_2-Cu, however the intensity of which was rather weak.** Such phenomenon could result from several factors:

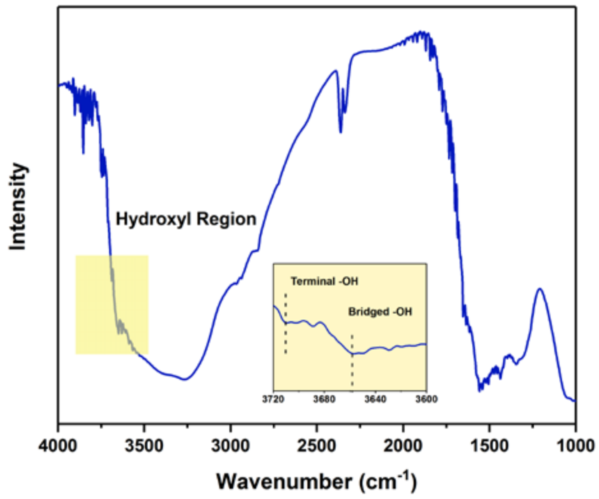

Figure R7 | DRIFTS results. *In-situ* DRIFTS spectra of inverse CeO_2/Cu under WGS conditions (at 200 °C) the inset figure was amplified OH region (3600–3800 cm^{-1}).

1. Composite of the catalysts. Our former work (Yan H. et al. *Appl. Catal. B*, 2018, 226, 182) has shown that for bulk catalysts containing large amount of Cu, the adsorption and OH stretching of H_2O were very weak.

2. Preparation method of the catalysts. In former report (Wang X. et al. *J. Phys. Chem. B*, 2006, 110, 428), the authors prepared Cu-CeO_2 catalysts with impregnation and reversed micro-emulsion method, respectively. The impregnated catalyst showed H_2O signal in the DRIFTS spectra, while the catalyst prepared by reversed micro-emulsion method did not. The reversed micro-emulsion method is applied to obtain bulk catalysts, which is similar to the AASA method used in our manuscript.

3. **Temperature.** In the same article (Wang X. et al. *J. Phys. Chem. B*, 2006, 110, 428), the impregnated catalyst showed obvious H_2O signal when the temperature reached 300 °C.

In our manuscript, using *in-situ* DRIFT, we focused more on the detection of the adsorbed CO. However, no adsorbed CO signal was detected, as shown in Figure R7, which might be due to the dark color of the catalyst powder.

To Reviewer #3:

Comment: *In this manuscript, the authors report on the preparation of an inverse catalyst consisting of rather large Cu nanoparticles that serve as a support for much smaller CeO_2 nanoparticles. Interestingly, such structures appear to show good activity and stability for the*

water-gas shift (WGS) reaction. With some small exceptions, the authors appear to have done a relatively thorough job in characterizing both the initial structure of the catalyst as well as the final structure under reaction conditions. (The final structure is very different from the initial structure, due to a very large extent of Cu sintering.). Overall, this concept of using relatively large Cu particles as supports for CeO_2 particles is an interesting one, though I do have a few comments for this manuscript:

Author Reply: We sincerely thank for the reviewer's kind comment.

Comment 1: *Did the authors determine the uncertainties based on replicate experiments for the results in Figure 1? It does not seem clear from the methods section. Showing meaningful uncertainty analysis is especially important for justifying the claim that the inverse CeO_2/Cu catalyst has higher activity at long times than does that commercial WGS catalyst. In general, there seems to be a lack of sufficient error analysis in the manuscript.*

Author Reply: The reviewer's comment is highly appreciated. Uncertainty analysis is crucial for the evaluation of catalytic performances. In fact, every catalyst shown in this work has been tested at least twice. Specially, we have prepared and tested the inverse CeO_2/Cu catalysts for three times. The tested catalysts were separately prepared before each measurement. As shown in Figure R8, the WGS activity was nearly the same. **According to the requirement of the reviewer, we have supplemented corresponding description in the experimental section (please see page 16, line 33–34) and Figure R8 was added as Supplementary Figure 1c in the revised manuscript.**

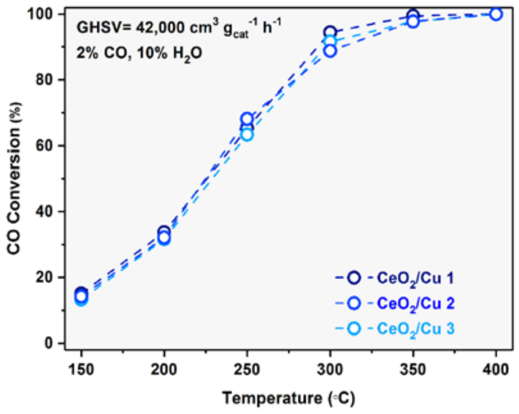

Figure R8 | Replicate measurements of WGS activity. Temperature-dependent activity of

WGS reaction on inverse CeO_2/Cu. (1atm total pressure, 100 mg of catalysts, 2% CO, 10% H_2O and the rest of N_2, GHSV = 42,000 $cm^3\ g^{-1}\ h^{-1}$)

Comment 2*: My other major comments is that the paper would be significantly improved if the authors could demonstrate that the reaction rate on their inverse system is proportional to some measure of the number of interfacial sites. Based on their characterization results and the cartoon pictured in Figure 1(h), can the authors estimate the interfacial areas for some of the catalysts shown in Supplementary figure 1, and perhaps demonstrate that the reaction rate scales with perimeter around the CeO_2 nanoparticles?*

Author Reply: We greatly thank for the reviewer's comment. As for the concern of quantified parameter, it was difficult because we cannot obtain useful information from TEM. **However, we have made efforts to obtain and compare the TOF between the inverse and normal catalysts, and the results were convincing.** First, we built a model for inverse CeO_2/Cu catalysts (Figure R5), with a CeO_2 nanoparticle (3 nm) on metallic Cu. The CeO_2 nanoparticle consisted of 231 Ce atoms. From this model, we found that there were 16 Ce atoms along the CeO_2-Cu periphery. **Thus, we could calculate the TOF of the single site on the periphery of CeO_2-Cu interface.**

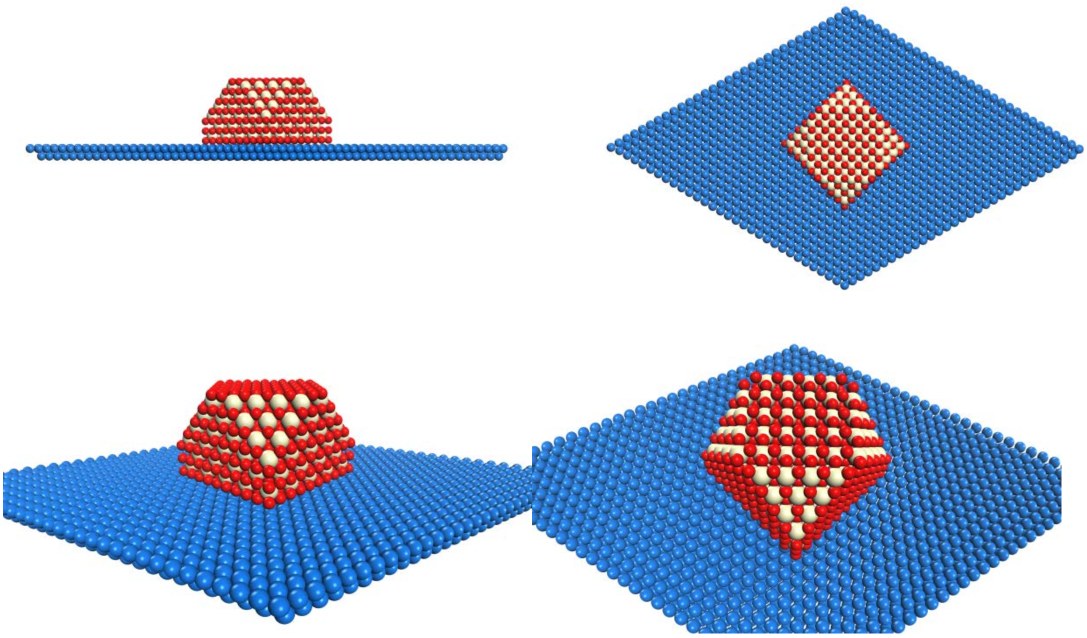

Figure R5 | Model of CeO_2 nanoparticle on Cu metal. The CeO_2 nanoparticle (~3 nm in size) contains 231 Ce atoms, and there are 16 Ce atoms along the Cu-CeO_2 periphery.

Based on this model, we calculated the TOF values of the single site on the periphery of CeO_2-Cu interface with the modified former formula (*J. Phys. Chem. C*, 2010, 114, 21605) :

$$TOF = \frac{r * N_{av} * m_{CeO_2}}{X_{CeO_2}} \frac{1}{16\pi d}$$

Where r was the reaction rate, N_{av} was Avogardro's constant, m_{CeO2} was the weight of the CeO_2 nanoparticle (assuming the semispherical shape, derived from its volume and density, 7.21 g cm^{-3}), X_{CeO2} was the CeO_2 concentration of the catalyst, 16 was the site density of Ce atoms along the periphery, and d was the crystalline size of CeO_2 (3 nm). **By using the above formula, the calculated TOF of inverse CeO_2/Cu was 0.058 s^{-1}.**

For the normal Cu/CeO_2 catalyst, we applied the formula below to obtain TOF of the single site on Cu-CeO_2 interface (*J. Phys. Chem. C*, 2010, 114, 21605):

$$TOF = \frac{r * Nav * mCu}{XCu} \frac{1}{8\pi d}$$

Where r was the reaction rate, N_{av} was Avogardro's constant, m_{Cu} was the weight of the Cu nanoparticle (assuming the semispherical shape, derived from its volume and density, 8.92 g cm^{-3}), X_{Cu} was the Cu concentration of the catalyst, 8 was the site density of Cu atoms along the periphery, calculated by π/2d(Cu-O), where d(Cu-O) was the length of Cu-O bond (0.195 nm), and d was the crystalline size of Cu (d ≈ 1/D, 1.3 nm). **By using the above formula, the calculated TOF of normal Cu/CeO_2 was 0.056 s^{-1}.**

The reaction rates (r) of the inverse CeO_2/Cu and normal Cu/CeO_2 were 47.3 and 10.2 μmol g^{-1} s^{-1}(Figure 1c). The amounts of interface sites (A) could be calculated with the formula:

$$A = \frac{r * Nav}{TOF}$$

Where r was the reaction rate and N_{av} was Avogardro's constant. **The calculated amount of interface sites for inverse CeO_2/Cu was 4.9 × 10^{20}, while that for normal Cu/CeO_2 was 1.1 × 10^{20}.**

The TOF values of the two catalysts were very close, indicating similar nature of the active sites. Besides, the amount of interface sites for inverse CeO_2/Cu was much larger, so we believed the promoted WGS activity of inverse catalyst originated from the enriched CeO_2/Cu interfaces. These important results were present as Supplementary

Scheme 1 and in Discussion part of the revised manuscript (please see page 14, line 10–12, line 17–23).

At the same time, we must note that the model for TOF calculations was simplified. The inverse catalyst exhibited more promising redox properties compared with the normal one, which has been shown from the *in-situ* Raman results (Figure R3). Therefore, the difference in activity between the inverse and normal catalyst could also come from the distinct ability in activating the reactants.

Comment 3*: It is a little unclear on page 5 when the authors report an activation energy of 37.1 kJ/mol for the inverse catalyst and that Cu-CeO$_2$ is 3.7 kJ/mol higher. The authors then say Cu-CeO$_2$ is expected to be close to 50-60 kJ/mol. Can they explain this apparent discrepancy?*

Author Reply: The reviewer's comment is highly appreciated. We have repeated the measurement of activation energy toward both catalysts for 3 times. The tested catalysts were separately prepared before each measurement. As shown in Figure R6, the *Ea* for the inverse CeO_2/Cu was from 36.9 to 37.3 kJ/mol, while that for normal Cu/CeO_2 was from 40.6 to 40.9 kJ/mol. **The repeated results suggested that the *Ea* of inverse CeO_2/Cu was ~4 kJ/mol lower than that of normal Cu/CeO_2. We have replaced Supplementary Figure 2 with Figure R6, and added corresponding discussion (please see page 6, line 17–20) of error in the revised manuscript.**

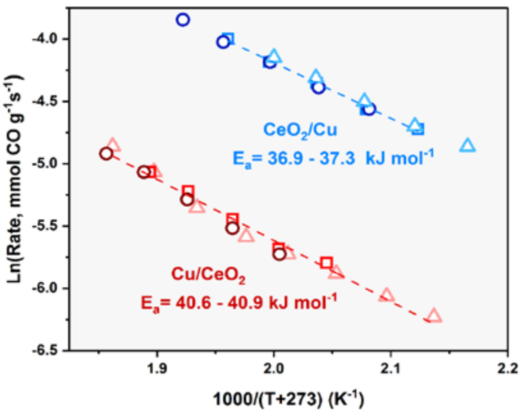

Figure R6 | Replicate measurements of activation energy. Arrhenius plots of WGS reaction on the inverse CeO_2/Cu catalyst and normal Cu/CeO_2 catalyst.

We have also gathered the *Ea* data of Cu-CeO_2 WGS catalysts. **As shown in Table R2 below, the E_a values could vary a lot with different preparation method and reaction atmosphere.** We are sorry for the saying that typical *Ea* of WGS on Cu-CeO_2 is 50~60 kJ mol^{-1}, which is not solid. **The discussion of E_a data has been modified (please see page 6, line 17–20) in the revised manuscript.**

Table R2 | Comparisons of WGS conditions, E_a and reaction rate of Cu-CeO_2 catalysts.

**Catalyst**	**Conditions**	**E_a/kJ mol^{-1}**	**Rate (200 °C)/ μmol g_{cat}^{-1} s^{-1}**
**$Cu_{0.9}Ce_{0.1}O_x$**	2%CO, 10%H_2O, balance N_2	37	8.9
**$Cu_{0.3}Ce_{0.7}O_x$**	2%CO, 10%H_2O, balance N_2	41	2.0
16 catalysts with different Cu-Ce ratio and method[1]	2%CO, 10%H_2O, balance He	34.2–59.5	0.2–3.0
8 wt.% Cu-CeO_2[2]	7%CO, 8.5% CO_2, 22%H_2O, 37% H_2, balance Ar	56	0.1
5at.% Cu-Ce(10% La)O_x[3]	2%CO, 10.7%H_2O, balance He	30.4	2.0
Inverse CeO_2/CuO[4]	1%CO, 3%H_2O, balance He	-	4.0

[1] Si R. et al. *Catal. Today* **2012**, *180*, 68.

[2] Koryabkina N.A. et al. *J. Catal.* **2003**, *217*, 233.

[3] Li Y. et al. *Appl. Catal. B* **2000**, *27*, 179.

[4] Barrio L. et al. *J. Phys. Chem. C* **2010**, *114*, 3580.

***Comment 4**: Is it surprising that the CeO_2 shows no chemical shifts as the Cu loading increases in Figure S4? Also, is there some reason that the Ce intensity does not appear to change over most of the range of bulk composition changes?*

Author Reply: We thank for the reviewer's kind comment. As pointed out by the reviewer, the fact that CeO_2 showing no chemical shift with increasing Cu was interesting. There are

two reasons of this phenomenon. First of all, most catalysts in this work, except the inverse CeO_2/Cu, contained more than 45 wt.% of CeO_2(Supplementary Table 1). With dominating amount of CeO_2, the Ce intensity in XPS could be similar (Figure S4), and the signal of CeO_2 interacting with Cu species could be overlapped by bulk CeO_2.

Moreover, for the inverse CeO_2/Cu, although the CeO_2 nanoparticles showed enhanced redox properties (evidenced by TPR and TPD), it could be easily oxidized in air, giving typical Ce^{4+} signals in *ex-situ* measurement (Figure 2d). We have also tried *ex-situ* XANES to examine the chemical state of Ce in inverse CeO_2/Cu. As shown in Figure R9, the result also gave nothing more than Ce^{4+} signal.

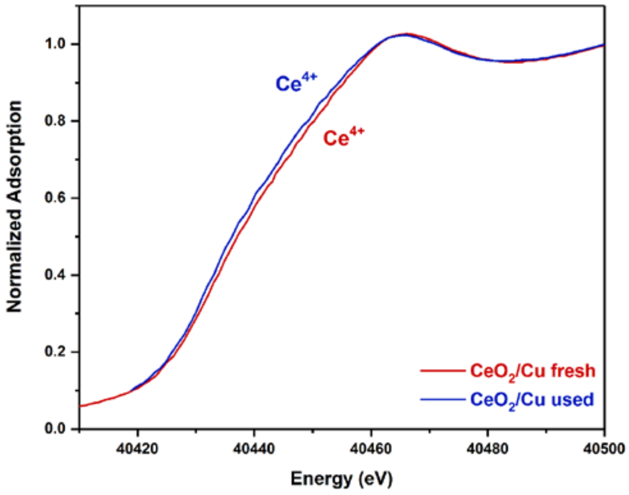

Figure R9 | XANES data. XANES spectra of fresh and used inverse CeO_2/Cu catalysts.

In order to check the chemical state of Ce under reaction conditions, we performed *in-situ* Raman experiments. We applied a similar procedure with TPSR, using cyclic CO and H_2O treatment under 200 °C. **As shown in Figure R2a,** two characteristic peaks, namely F_{2g} peak of CeO_2[2] and surface defect (oxygen vacancy) peak *of D* (Guo, M. et al. *Langmuir*, 2011, 27, 3872), could be observed. **It is surprising that when CO was introduced, the defect peak (*D*) of inverse CeO_2/Cu was greatly pronounced, even exceeding the CeO_2 F_{2g} peak.** When H_2O was introduced, the defect peak was clearly weakened. This meant that the introduction of CO induced the creation of surface vacancies through the reaction of CO + O $\rightarrow$ CO_2 and CO + –OH $\rightarrow$ CO_2 + 1/2H_2. The normal Cu/CeO_2 went through the same experiment (Figure R2b), showing apparently fewer defect sites. These results suggested that for inverse CeO_2/Cu, the surface defect sites related to Ce^{3+} were easily generated under WGS

conditions. Also the strong ability of H_2O association catalyzed by the surface defects of CeO_2 ensured the very high WGS activity. **These important data were added in the revised manuscript as Figure 5 and relevant discussion was supplemented (please see page 12, line 3–7, page 13, line 1–10).**

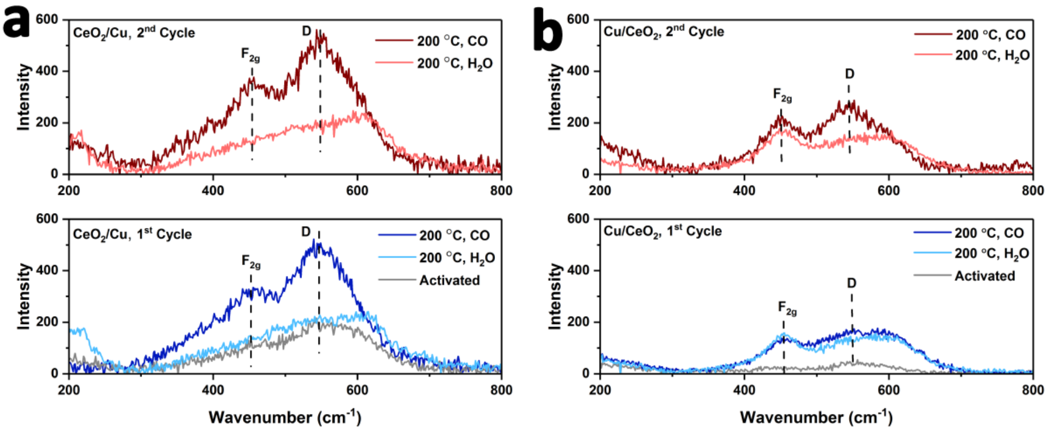

Figure R2 | Defect sites of the catalysts. *In-situ* Raman spectra of (a) inverse CeO_2/Cu and (b) normal Cu/CeO_2 catalysts (633-nm laser).

In-situ Raman experiments were also conducted under the WGS conditions. As shown in Figure R3a, the inverse CeO_2/Cu also gave more pronounced signal of surface defects under the WGS conditions. However, the *in-situ* Raman results of pure CeO_2 gave a dominant CeO_2 F_{2g} peak with very strong intensity (Figure R3b), which was quite different to that for the inverse CeO_2/Cu catalyst. The quantified results showed that the inverse CeO_2/Cu possessed abundant defect sites under WGS conditions, with D/F_{2g} larger than 1.6. These results also suggested that defect sites (Ce^{3+}) were very easily generated under the WGS reaction conditions. We have measured the H_2O reaction orders of the catalysts (Supplementary Figure 11), those were between 0.20–0.27, much lower than those of the CO (0.75–0.77). These results demonstrated the strong ability of consuming H_2O on the inverse catalysts, which was well consistent the above *in-situ* Raman result. **Thus, we concluded that the interface of bulk Cu – nano CeO_2 greatly enhanced the redox properties of the catalyst. These important results were added in the revised manuscript as Figure 5c and relevant discussion was supplemented (please see page 10, line 1–11, page 11, line 1–9).**

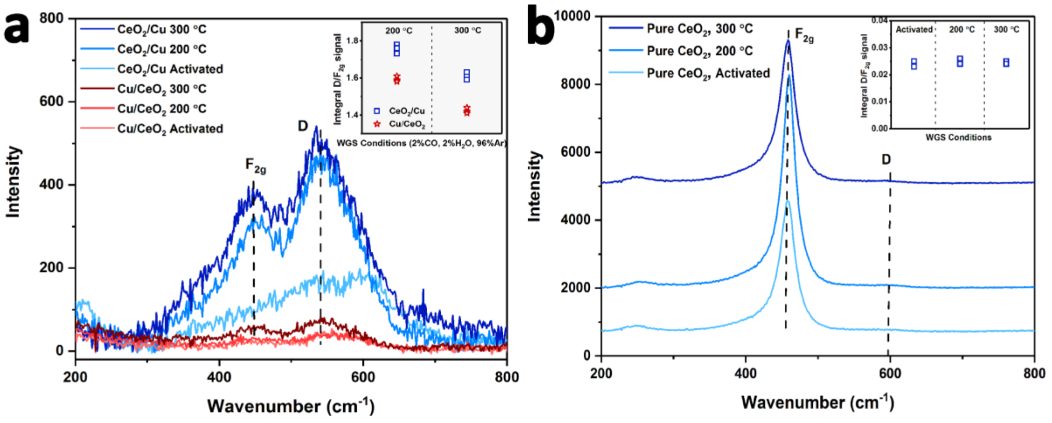

Figure R3 | Defect sites of the catalysts. *In-situ* Raman spectra under WGS conditions of (a) inverse CeO_2/Cu and normal Cu/CeO_2 catalysts and (b) pure CeO_2; the inset Figures give statistical results of the D/F_{2g} integral intensity ratio.

Comment 5*: Can the authors comment on how the Cu surface areas / particle sizes compare between the catalyst reported here, the supported Cu/CeO_2 catalyst, and the Cu-Zn-Al catalyst?*

Author Reply: We thank for the reviewer's comment. We have found that the inverse CeO_2/Cu catalyst shows surprisingly high WGS activity, considering its high Cu content. According to the reviewer's concern, we calculated the Cu surface areas, and the reaction rates in terms of Cu surface area and Cu loading.

Table R1 | Cu content, BET and Cu Surface areas and Cu dispersion.

**Catalyst**	**Cu wt.%** [a]		**S_{BET}/m^2 g^{-1}**		**S_{Cu}/m^2 g^{-1}**	**Cu dispersion**[c]
	**Fresh**	**Used**	**Fresh**	**Used**		
CeO_2/Cu ($Cu_{0.9}Ce_{0.1}O_x$)	61.5	82.9	47.7	16.2	**50.7**	9.1%
Cu/CeO_2 ($Cu_{0.3}Ce_{0.7}O_x$)	10.7	13.4	46.5	42.8	**71.7**	79.6%

As shown in Figure R4a, when the reaction rates were calculated with Cu loading, the normal Cu/CeO_2 gave higher *r* than inverse CeO_2/Cu since it contained much less Cu. **In our**

manuscript, we found that the active site for the inverse catalyst lied in the Cu-CeO_2 interface, rather than metallic Cu. With this premise, the WGS reaction rate would not be accurate in terms of Cu loading. Moreover, the calculated S_{Cu} for inverse CeO_2/Cu and normal Cu/CeO_2 were larger than their S_{BET}. Such phenomenon has been reported in previous literatures (For example, Cu-Zn-Al in Velu S. et al. *Catal. Lett.* 1999, 62, 159). We have mentioned in the manuscript that CeO_2 was found to participate in N_2O chemisorption, causing higher Cu dispersion (Wang W. et al. *ACS Catal*. 2017, 7, 1313–1329). **We calculated the Cu surface area of Cu-Zn-Al catalyst (19.7 m^2 g^{-1}), which was much lower than that of inverse CeO_2/Cu.** Thus, though the reaction rate of inverse CeO_2/Cu was higher than that of normal Cu/CeO_2 (Figure R4b), the accuracy of r derived from S_{Cu} could not be ensured in this system. This is the reason we did not put these data in our manuscript.

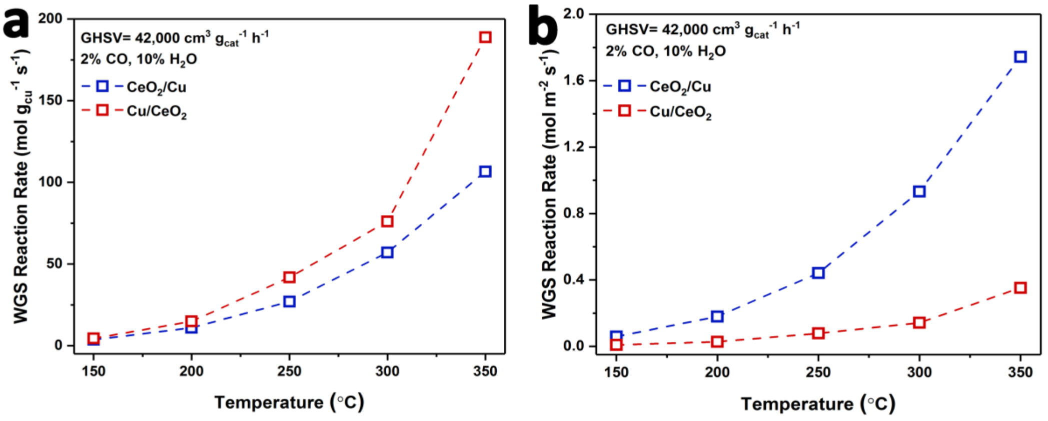

Figure R4 | WGS reaction rates with different calculations. (a) Reaction rates of inverse CeO_2/Cu and normal Cu/CeO_2 in terms of Cu loading; (b) Reaction rates of inverse CeO_2/Cu and normal Cu/CeO_2 in terms of Cu surface area. (1atm total pressure, 100 mg of catalysts, 2% CO, 10% H_2O and the rest of N_2, GHSV = 42,000 cm^3 g^{-1} h^{-1})

Comment 6*: Finally, while the results are clearly communicated, there are numerous grammatical errors in the manuscript*

Author Reply: We greatly thank for the reviewer. **The manuscript has been thoroughly checked and many writing errors are found and corrected.** For example, "CO reduction" in abstract and introduction was corrected to "CO oxidation". The unit in experimental section was unified as cm^3 min^{-1} rather than mL min^{-1}.

REVIEWERS' COMMENTS:

Reviewer #1 (Remarks to the Author):

The authors have significantly change the original manuscript in response to the original comments, adding new information/experiments, revising their assumptions and conclusions about the mechanism and clarifying overall all the suggested points. I now recommend publication.

Reviewer #3 (Remarks to the Author):

The authors have done a thorough job responding to the reviews, and I find that the manuscript is significantly improved and worthy of publication. I have only one small final question / comment: in discussing Figure 4, the authors write that "After H_2O injection, we removed the surface oxygen with H_2 reduction and preserved the surface hydroxyls, after which CO was purged into the system to conduct the TPSR test again. " How did they determine that the surface oxygen was removed but that the hydroxyls coverage was preserved?

Responses to the reviewers' comments

To Reviewer #1:

Comment: *The authors have significantly change the original manuscript in response to the original comments, adding new information/experiments, revising their assumptions and conclusions about the mechanism and clarifying overall all the suggested points. I now recommend publication.*

Author Reply: We greatly thank for the reviewer. Your comments have significantly helped us improve the mechanism study. Thanks for your kind comments.

To Reviewer #3:

Comment: *The authors have done a thorough job responding to the reviews, and I find that the manuscript is significantly improved and worthy of publication. I have only one small final question / comment: in discussing Figure 4, the authors write that "After H2O injection, we removed the surface oxygen with H2 reduction and preserved the surface hydroxyls, after which CO was purged into the system to conduct the TPSR test again. " How did they determine that the surface oxygen was removed but that the hydroxyls coverage was preserved?*

Author Reply: We sincerely thank for the reviewer's kind comment. In our manuscript, we have found that the surface oxygen of inverse CeO_2/Cu is easy to be removed. The surface oxygen can react with either CO (in WGS conditions) or H_2 (in pretreatment). Meanwhile, CO can react with surface hydroxyls, following the reaction:

$$CO + OH \rightarrow CO_2 + 1/2H_2$$

Such reaction can be observed from Fig. 4a and b. When CO was introduced, the intensity of H_2 signal was increased. The consumption of surface hydroxyls generates H_2, so the reaction of H_2 with surface hydroxyls is not likely to occur.

Thus, we use H_2 treatment to remove surface oxygen and preserve surface hydroxyls. We have also conducted such experiments in our former work (Fu X. et al. *J. Am. Chem. Soc*. 2019, 141, 4613).
